# DUX4 Binding to Retroelements Creates Promoters That Are Active in FSHD Muscle and Testis

**DOI:** 10.1371/journal.pgen.1003947

**Published:** 2013-11-21

**Authors:** Janet M. Young, Jennifer L. Whiddon, Zizhen Yao, Bhavatharini Kasinathan, Lauren Snider, Linda N. Geng, Judit Balog, Rabi Tawil, Silvère M. van der Maarel, Stephen J. Tapscott

**Affiliations:** 1 Division of Human Biology, Fred Hutchinson Cancer Research Center, Seattle, Washington, United States of America; 2 School of Medicine, University of Washington, Seattle, Washington, United States of America; 3 Department of Human Genetics, Leiden University Medical Center, Leiden, The Netherlands; 4 Department of Neurology, University of Rochester Medical Center, Rochester, New York, United States of America; University of Utah School of Medicine, United States of America

## Abstract

The human double-homeodomain retrogene *DUX4* is expressed in the testis and epigenetically repressed in somatic tissues. Facioscapulohumeral muscular dystrophy (FSHD) is caused by mutations that decrease the epigenetic repression of *DUX4* in somatic tissues and result in mis-expression of this transcription factor in skeletal muscle. DUX4 binds sites in the human genome that contain a double-homeobox sequence motif, including sites in unique regions of the genome as well as many sites in repetitive elements. Using ChIP-seq and RNA-seq on myoblasts transduced with *DUX4* we show that DUX4 binds and activates transcription of mammalian apparent LTR-retrotransposons (MaLRs), endogenous retrovirus (ERVL and ERVK) elements, and pericentromeric satellite HSATII sequences. Some DUX4-activated MaLR and ERV elements create novel promoters for genes, long non-coding RNAs, and antisense transcripts. Many of these novel transcripts are expressed in FSHD muscle cells but not control cells, and thus might contribute to FSHD pathology. For example, *HEY1*, a repressor of myogenesis, is activated by DUX4 through a MaLR promoter. DUX4-bound motifs, including those in repetitive elements, show evolutionary conservation and some repeat-initiated transcripts are expressed in healthy testis, the normal expression site of *DUX4*, but more rarely in other somatic tissues. Testis expression patterns are known to have evolved rapidly in mammals, but the mechanisms behind this rapid change have not yet been identified: our results suggest that mobilization of MaLR and ERV elements during mammalian evolution altered germline gene expression patterns through transcriptional activation by DUX4. Our findings demonstrate a role for DUX4 and repetitive elements in mammalian germline evolution and in FSHD muscular dystrophy.

## Author Summary

Transposable elements (TEs) are found in most genomes, and many TEs create extra copies of themselves in new genomic locations by a process called retrotransposition. TEs are often thought of as genomic parasites that must be suppressed, because retrotransposition can cause great harm to their host organism. However, during evolution, the functions encoded by TEs have sometimes been co-opted to the advantage of the host genome as novel genes or as gene regulatory regions. We studied a human transcription factor called *DUX4* that is normally expressed in testis and repressed in muscle. Sometimes muscle repression fails, causing the disease facioscapulohumeral muscular dystrophy (FSHD). We find that DUX4 binds many TE types and can activate their transcription. Some activated TEs have been co-opted as novel promoters for human genes. DUX4's activation of these genes via TEs might be important in the biology of normal testis and may contribute to the FSHD disease process. Our findings raise the possibility that DUX4 and TEs co-evolved, as TEs may have hijacked DUX4 to aid their retrotransposition while DUX4 may have utilized TEs to modify its transcriptional network in the evolving germline.

## Introduction

The transcription factor *DUX4* is a member of a small family of double-homeodomain genes that also includes paralogs *DUXA*, *DUXB*, *DUXBL*, *DUXC* and murine *Dux* [1]–[3]. The primate-specific *DUX4* gene likely arose from retrotransposition of the *DUXC* mRNA, with subsequent deletion of the *DUXC* gene from the primate genome [3]. *DUX4* exists in the primate genome as part of a ∼3.3 kb repeat unit called D4Z4, found in macrosatellite arrays in the subtelomeric regions of human chromosomes 4 and 10. Poorly studied arrays are also found in other genomic regions and appear to contain interrupted versions of the *DUX4* ORF [4], [5]. The number of D4Z4 units in each array varies between human individuals [6], and array size and number of genomic loci vary between different primate species [7].

*DUX4* is expressed in germ-line cells of the testis and is epigenetically repressed in somatic tissues [8]–[11], likely in part through repeat-mediated repression [12]. Deletions of the D4Z4 array to fewer than 10 repeat units or mutations in *SMCHD1*, a gene necessary for repeat-mediated epigenetic repression, result in decreased epigenetic repression of *DUX4* in skeletal muscle, causing a human muscle disease, facioscapulohumeral muscular dystrophy (FSHD; OMIM #158900, #158901) [6], [9], [13]–[15]. The decreased epigenetic repression results in occasional bursts of *DUX4* expression in a subset of nuclei in FSHD muscle cells [9], [16], which appears to cause death of expressing muscle cells [17]–[19]. Our prior work showed, using microarrays and ChIP-seq, that mis-expression of *DUX4* in human skeletal muscle activates the expression of many genes normally expressed in the germline and that DUX4 binds a double-homeobox motif at ∼60,000 sites in the mappable genome [10]. Although many DUX4 binding sites overlap non-repetitive regulatory elements of its activated genes, more than half of the ChIP-seq peaks overlap repeat elements of the MaLR class [10].

MaLRs, or mammalian apparent LTR-retrotransposons, are distantly related to endogenous retroviruses (ERVs), and comprise ∼3.7% of the human genome with ∼350,000 copies of various subclasses of MaLR elements [20]–[23]. Like ERVs, MaLRs are selfish elements that spread in the genome by a copy-paste mechanism called retrotransposition (transcription, reverse transcription and reintegration). ERVs and MaLRs have a pair of long-terminal repeats (LTRs) that act as strong promoters and that flank one or more open reading frames (ORFs). Post-insertion deletions mediated by homology between the two LTRs of an ERV or MaLR element often leave single (or “solo”) LTR elements in the genome. The internal ORFs of conventional ERVs encode the gag, pro, pol, and sometimes env proteins needed for replication and re-integration, whereas the single MaLR ORF encodes a protein with an ∼90-aa stretch of homology to the ERVL gag protein, suggesting that MaLRs derived from ERVL-like retrotransposons [24]. The homology to the ERVL gag protein and the absence of the other proteins necessary for ERV replication suggest that MaLRs might rely on concomitantly expressed ERVs to provide these other proteins. Most human ERV and MaLR elements inserted into the primate genome before the divergence of Old and New World monkeys and are generally thought to be no longer capable of retrotransposition (unlike in rodents, where ERVs and MaLRs are still active). However, reports of very rare polymorphic ERV and MaLR insertions in humans suggest that occasional transposition events still occur in the human population [25], [26].

Retrotransposition provides a way to spread regulatory sequences to large numbers of new genomic locations in short evolutionary time. Some families of repetitive elements appear to have been involved in large-scale rewiring of transcriptional networks [27], [28] and a number of cellular transcription factors have been shown to bind repetitive elements [29]–[31]. Repeats, especially LTRs, can be co-opted (or “exapted”) to act as promoters for genes of the host genome [32], [33]. For example, when placentation evolved during early mammalian divergence, a large number of genes acquired expression in endometrial cells via upstream insertion of the eutherian-specific MER20 transposon [34]. In another example, binding of the OCT4 and NANOG transcription factors to various primate-specific repeats explains many of the differences observed between the transcriptional networks of human and mouse ES cells [31]. To date, no such repeat-mediated rewiring of the male germ cell transcriptional network has been reported, yet testis expression patterns are known have evolved rapidly in mammals [35].

To determine whether DUX4 binding to repetitive elements can affect transcriptional networks, we analyzed ChIP-seq and RNA-seq datasets in skeletal muscle cells that ectopically express *DUX4*, as well as RNA-seq data from FSHD patient and control muscle cells. We find that repetitive elements are bound and activated by DUX4, including primate-specific MaLR and ERV LTRs, and that some full-length repeat elements are transcribed in response to DUX4. In addition, some DUX4-bound LTRs form novel first exons of annotated genes, long-noncoding (lnc) RNAs, and antisense RNAs. Together, our findings demonstrate that DUX4's transcriptional network is mediated in part through binding to repetitive elements, including some primate-specific retroelements that could contribute to lineage-specific patterns of gene expression [35].

## Results

### DUX4 binds LTR elements, especially of the MaLR family

**Figure 1:**
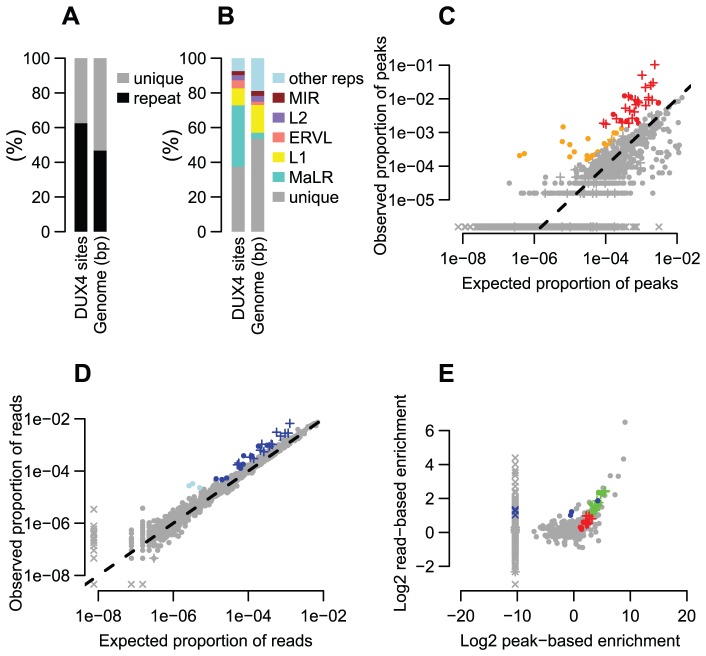
Many repeat types are enriched among DUX4 binding sites. (A) ∼2/3 of DUX4 binding-sites are in repetitive elements, compared to ∼45% of the human genome. (B) Comparing repeat family proportions among DUX4 binding-sites with genome-wide fractions shows ∼10-fold MaLR enrichment. (C) A peak-based method of estimating repeat enrichment uses uniquely-mapped reads, so is blind to recently active repeats; however, it ignores background reads so provides a more sensitive enrichment measure than the read-based estimate (Figures 1D, 1E). 32 repeat types (red) are enriched ≥2-fold with ≥100 peaks (arbitrary thresholds) (Tables 1, S2 and S3); 21 (orange) are rarer in the genome (10–99 peaks) but enriched ≥4-fold. The log10-scaled x-axis shows the proportion of peaks expected to overlap each repeat type if DUX4 binding sites had uniform genomic distribution; the log10-scaled y-axis shows observed proportions. The dashed line represents no enrichment. In all panels, “+” symbols represent MaLR elements and “x” datapoints represent repeat types for which no peaks/reads were observed – these are given an arbitrary low (non-zero) value to ensure visibility on log-scaled plots. (D) The read-based enrichment estimation method examines highly similar repeats as well as uniquely-mappable sequences, but gives a “dampened” enrichment measure due to background reads in ChIP-seq samples (see Methods). 25 repeat types (dark blue) are enriched ≥2-fold, with ≥1000 reads (arbitrary thresholds); 3 (light blue) are rarer among ChIP-seq reads (100–999 reads) but enriched ≥4-fold. (E) The peak-based (x-axis) and read-based (y-axis) methods yield similar results. 19 repeat types (green datapoints, thresholds as in Figures 1A and B for red and dark blue points) were enriched in both analyses; 13 enriched only by the peak-based method (red), and 6 enriched only by the read-based method (blue). Additional repeats (gray datapoints, upper-right quadrant) appear enriched by both methods, but are rare in the genome so do not exceed our arbitrary peak/read thresholds.

In previously published work, we observed that many of the ∼60,000 DUX4 binding sites identified by ChIP-seq following expression of *DUX4* in human myoblasts overlap MaLR LTR elements [10]. Using a more recent version of the human genome assembly (hg19) and including the X and Y chromosomes, we identified 63,795 DUX4 binding-sites (Table S1). Overall, ∼2/3 of sites are in a repetitive element, more than expected given that ∼45% of the human genome is recognizable as repeats (Figure 1A) [21]. Furthermore, ∼1/3 of DUX4 binding sites are in a MaLR element, ∼10-fold more than would be expected if binding sites were dispersed randomly throughout the genome (Figure 1B). Because DUX4 binds an AT-rich sequence motif, its binding sites might show repeat enrichment based simply on GC content; however, when we randomly sampled genomic locations with similar AT-content to the DUX4 binding site (see Methods), we find that only ∼49% overlapped repeats of any class, and only ∼3% overlapped MaLR elements. In order to determine which subclasses of MaLRs are responsible for the enrichment, and to explore which other repetitive elements might also bind DUX4, we systematically analyzed ChIP-seq data using two complementary approaches.

An analysis of repetitive element content among DUX4 ChIP-seq peaks shows that enrichment is heavily biased towards LTR elements (Figure 1, Table 1, S2 and S3), particularly those of the MaLR class: of 32 enriched repeat types (using arbitrary thresholds of ≥2-fold enrichment and ≥100 DUX4-bound instances), 30 are in the LTR class, and of those, 23/30 are MaLRs. Many subtypes of MaLR-LTRs contribute to the MaLR family-wide enrichment, including the LTRs of THE1 elements (A, B, C and D subfamilies) that were active early in the primate lineage [20] and MLT1 subtypes that were active before mammalian radiation. Outside of the MaLR family, other LTR subtypes also show enrichment, including primate-specific ERVL-LTRs (MLT2A1 and MLT2A2) and hominoid-specific ERVK-LTRs (MER11B and MER11C) (Table 1). Some non-LTR repeats are also enriched among DUX4 binding sites, including the HSAT5 and HSATII satellite repeats and some simple repeats like the (CAAT)n tetranucleotide repeat, although with fewer mappable DUX4 binding sites (<100 DUX4-bound instances). In almost all cases, the consensus sequences for enriched repeats contain at least one DUX binding motif (Figure S1). Analysis of ENCODE ChIP-seq data for 161 other regulatory factors (see Methods) shows that these DUX4-bound regions are not generally bound by other transcription factors (the factor with most overlapping peaks, Runx3, only bound 0.9% of DUX4-bound repeat regions).

**Table 1:** Highly enriched repeat types among DUX4 binding sites.

repeat type^1^	repeat family	num. repeats in genome	num. ChIP-seq peaks	percent genomic repeat instances bound	peak-based ChIP-seq enrichment estimate	read-based ChIP-seq enrichment estimate	read-based RNA-seq activation estimate	num. bound activated regions	percentage bound regions showing activation
THE1C	ERVL-MaLR	9,874	3,214	32.9%	**48.3×**	**5.4×**	3.8×	43	1.3%
THE1B	ERVL-MaLR	22,433	6,594	29.6%	**43.6×**	**5.2×**	3.8×	65	1.0%
MER11B	ERVK	548	220	40.1%	**20.9×**	**5.1×**	7.3×	0	0.0%
MLT2A1	ERVL	3,780	778	20.7%	**36×**	**4.8×**	25×	42	5.4%
THE1A	ERVL-MaLR	4,233	742	17.7%	**26.8×**	**4.7×**	1.5×	16	2.2%
HSAT5	Satellite	260	12	5.0%	**19.1×**	**3.7×**	4.2×	0	0.0%
MLT1E1	ERVL-MaLR	1,043	112	10.7%	**17.2×**	**3.6×**	2.5×	1	0.9%
MLT1E	ERVL-MaLR	908	120	13.3%	**21.6×**	**3.4×**	3.9×	4	3.3%
MER11C	ERVK	866	138	16.4%	**8×**	**3.2×**	1.4×	1	0.7%
MLT2A2	ERVL	3,898	733	18.9%	**22.9×**	**3.1×**	3.1×	8	1.1%
THE1D	ERVL-MaLR	12,642	1,386	11.0%	**16.7×**	**3×**	3.1×	38	2.7%
MLT1E1A	ERVL-MaLR	3,362	337	10.1%	**14.7×**	**3×**	2.9×	10	3.0%
MLT1E3	ERVL-MaLR	2,038	164	8.7%	**12.9×**	**3×**	24.9×	4	2.4%
MLT1A0	ERVL-MaLR	20,643	1,609	7.9%	**13.7×**	**2.9×**	1.2×	25	1.6%
MLT1F2	ERVL-MaLR	6,036	600	10.1%	**14×**	**2.6×**	1.6×	2	0.3%
GC_rich	Low_complexity	13,724	0	0.0%	**0×**	**2.5×**	0.9×	0	0.0%
MLT1D	ERVL-MaLR	20,741	1,914	9.3%	**14.1×**	**2.5×**	2.1×	33	1.7%
LSAU	Satellite	129	0	0.0%	**0×**	**2.4×**	0.3×	0	0.0%
MLT1A	ERVL-MaLR	9,070	549	6.1%	**10.8×**	**2.4×**	3.6×	12	2.2%
MSTD	ERVL-MaLR	7,665	426	5.6%	**9.5×**	**2.4×**	1.5×	3	0.7%
(CCA)n	Simple_repeat	1,222	2	0.2%	**0.7×**	**2.3×**	1.2×	0	0.0%
MSTC	ERVL-MaLR	3,169	125	4.0%	**6.8×**	**2.2×**	1×	1	0.8%
MLT1E2	ERVL-MaLR	3,996	289	7.4%	**9.7×**	**2.1×**	3.8×	12	4.2%
SVA_F	Other	1,025	0	0.0%	**0×**	**2×**	2.8×	0	0.0%
LTR2B	ERV1	326	2	0.6%	**0.7×**	**2×**	0.8×	0	0.0%
MSTA	ERVL-MaLR	19,782	588	3.0%	**4.6×**	**2×**	2.8×	1	0.2%
MLT1A1	ERVL-MaLR	6,766	261	3.9%	**6.4×**	**1.9×**	2×	6	2.3%
MLT1B	ERVL-MaLR	18,004	693	3.9%	**6.9×**	**1.8×**	1.6×	6	0.9%
MLT1L	ERVL-MaLR	12,074	484	4.0%	**9.1×**	**1.8×**	1.3×	7	1.4%
MLT2B3	ERVL	3,313	142	4.3%	**5.7×**	**1.5×**	0.9×	0	0.0%
MER21C	ERVL	5,501	153	2.8%	**3.2×**	**1.5×**	0.6×	0	0.0%
MLT1F	ERVL-MaLR	4,297	119	2.8%	**4.2×**	**1.5×**	1.4×	2	1.7%
MLT1K	ERVL-MaLR	18,173	402	2.2%	**4.7×**	**1.5×**	1.1×	0	0.0%
THE1B-int	ERVL-MaLR	4,230	594	14.2%	**5.8×**	**1.4×**	6.6×	16	2.7%
THE1A-int	ERVL-MaLR	1,464	168	11.7%	**4.7×**	**1.4×**	1.8×	7	4.2%
ERVL-B4-int	ERVL	3,883	119	3.1%	**2.2×**	**1.2×**	1.5×	0	0.0%
L1MC4	L1	29,614	492	1.7%	**2.6×**	**1.2×**	1.2×	5	1.0%
Tigger1	TcMar-Tigger	12,109	480	4.0%	**2.5×**	**1.1×**	1.6×	2	0.4%

1 We show only repeat types with ≥2-fold enrichment among either the ChIP-seq peaks (showing only those with ≥100 peaks) or among the ChIP-seq reads (showing only those with ≥1000 reads). Repeat types are shown sorted by read-based enrichment estimate. Full results for all repeat types are shown in Table S2, and are given aggregated by repeat family in Table S3.

It remains challenging to accurately map sequence reads to repetitive elements with high sequence identity [36], and the peak-based ChIP-seq analysis we described above is relatively blind to recently mobilized repeats because it only uses sequence reads that map uniquely in the human genome. Therefore we also used a complementary approach, adapting a previously published method [37] that estimates enrichment regardless of whether individual ChIP-seq reads map uniquely in the genome (Figure 1D, Methods). The method aggregates read counts over all genomic copies of each repeat class rather than trying to map reads uniquely to individual repeat instances. Read counts for a test sample are then compared with counts for a control sample to determine enrichment. Although this read-based method can look at recently mobilized repeats, it gives a “dampened” measure of repeat enrichment due to background reads in ChIP-seq samples and is therefore less sensitive to modest levels of enrichment (see Methods). This read-based method identified a similar set of repetitive elements as the peak-based method (Figure 1E), and, in addition, revealed enrichment of a small number of repeat types, such as the SVA_F subfamily, that were not detected using the peak-based method (Figure 1E, Tables 1, S2 and S3). SVAs are composite retroelements that include segments derived from SINE, VNTR and Alu repeats, and have been very recently active in the human, chimpanzee and gorilla genomes [38].

**Figure 2:**
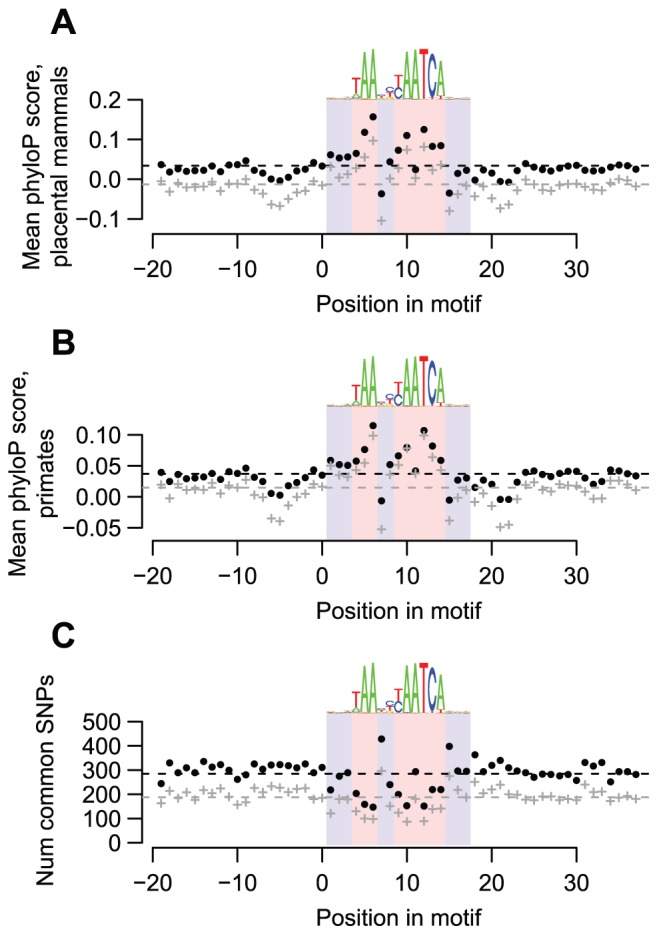
DUX4-bound motifs are evolutionarily conserved. (A) DUX4 motifs are conserved among placental mammals. We obtained phylogenetic conservation scores for placental mammals (phyloP scores) via the UCSC genome browser for the top-scoring DUX4 motif in each of the 63,795 DUX4 ChIP-seq peaks, along with scores for 20 bp flanking regions on each side. For each position in the motif, we show the mean phyloP score across all 63,795 motifs (black datapoints) or across just motifs overlapping repetitive elements (gray datapoints). The dashed lines show overall mean scores for the entire motif plus their flanking regions. We use blue and red background shading to indicate the 17 nucleotide positions that comprise the DUX4 motif, with light red shading showing the least variable nucleotide positions of the motif. A sequence logo of the motif is shown above each graph. It is clear that most of the nucleotide positions that are least variable in the DUX4 motif have higher conservation scores than the surrounding positions. (B) DUX4 motifs are conserved among primates. Primate phyloP scores, plotted as in panel A. (C) Common SNPs are under-represented in DUX4-bound motifs. We used the UCSC genome browser to determine the locations of common SNPs (≥1% minor allele frequency) within any of the 63,795 DUX4-bound motifs and their flanking regions. We plot the number of SNPs found at each position in any of the 63,795 DUX4-bound motifs (black datapoints), or in just motifs overlapping repetitive elements (gray datapoints). It is clear that there are fewer SNPs in most of the nucleotide positions that are least variable in the DUX4 motif than in the surrounding positions.

To examine whether DUX4 binding sites are functionally important, especially those in repetitive elements, we determined whether they are evolutionarily conserved. We find that DUX4-bound motifs have tolerated fewer sequence changes than flanking sequences during the evolution of placental mammals (Figure 2A), primates (Figure 2B), and humans (Figure 2C). These findings hold true even if we consider only motifs in repetitive sequences (Figure 2, gray datapoints), demonstrating that at least a subset of DUX4 binding sites in repetitive elements are conserved.

### DUX4 activates transcription from bound repetitive elements

**Figure 3:**
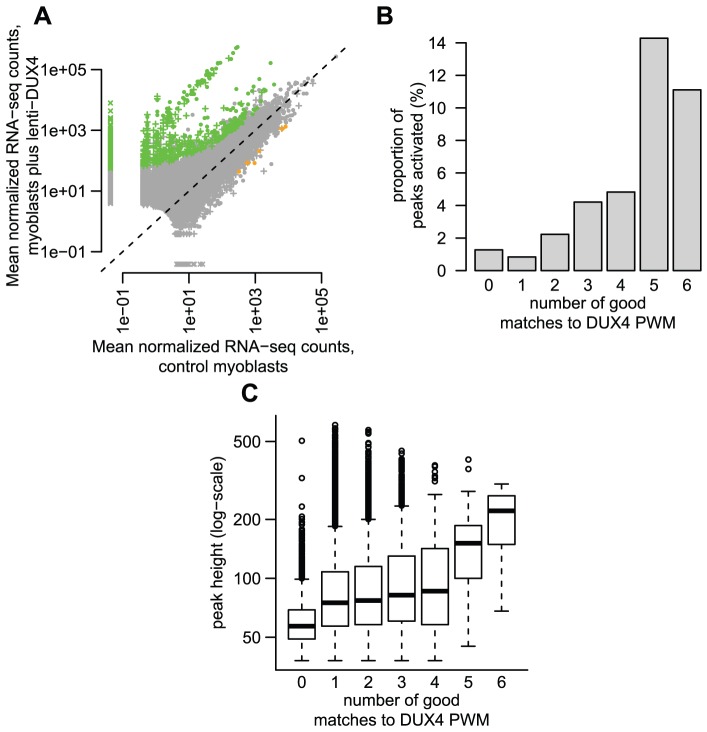
Some DUX4-bound repetitive elements are transcriptionally activated by DUX4. (A) ∼1% of DUX4-bound regions show statistically-significant activation in response to DUX4 in our conservative analysis. We show normalized RNA-seq read counts within an arbitrary distance of 1 kb from DUX4-bound regions (peaks), comparing counts averaged over two myoblast lines that do not express *DUX4* (x-axis) to counts averaged over two myoblast lines transduced with lentiviral *DUX4* (y-axis). Only regions with at least 10 reads summed over all four samples are shown, and counts are plotted on a log scale. The 738 regions shown as green points show statistically significant activation in response to DUX4 (≥2-fold activation, FDR-adjusted p-value≤0.1) and the 6 orange points show significant repression (same thresholds). Regions surrounding DUX4-bound repeats are shown as “+” symbols, with dots representing DUX4-bound unique regions. Some regions (“x” symbols) had normalized counts of 0 in one condition and are plotted at an arbitrary low value so they appear on a log-scale. (B) DUX4-bound regions with more predicted DUX4 binding motifs are more likely to be transcriptionally activated. The y-axis value gives the percentage of regions that are transcriptionally activated by DUX4 (≥2-fold, FDR-adjusted p-value≤0.1). (C) DUX4-bound regions with more predicted DUX4 binding motifs have greater DUX4 occupancy, using ChIP-seq peak height as a proxy for DUX4 occupancy (shown on a log-scale).

In order to determine whether DUX4 binding to repetitive elements results in their transcriptional activation, we generated RNA-seq data (100 nt single-end reads) from *DUX4*-transduced and control myoblasts. In a conservative analysis, we identified 738 DUX4-activated transcripts within 1 kb on either side of a DUX4 ChIP-seq peak and six DUX4-repressed transcripts (Figure 3A, ≥2-fold change, FDR-adjusted p-value<0.1). These 738 bound and activated regions comprise 1.2% of the 63,795 DUX4-bound sites. Peaks with multiple DUX4-binding sites are more likely to initiate transcripts than those with a single motif (Figure 3B; 2.5% of peaks with more than one site were associated with lenti-*DUX4*-transcriptional induction, compared to only ∼0.8% peaks with 1 motif; p<10^−15^, chi-squared test). Peaks with more than one motif also show greater ChIP-seq peak height, a proxy measurement for DUX4 occupancy (Figure 3C).

DUX4-bound repetitive elements are just as likely to initiate a transcript as DUX4 binding sites in unique sequence (∼1.2% of both classes show activation), with 454/738 (∼62%) of DUX4-initiated transcripts arising in or near a repetitive element. The DUX4-bound repeat types most likely to be transcribed are HSATII elements (23% of bound HSATII repeats show activation) and MLT2A1 ERVL-LTRs (5.4% of bound MLT2A1 LTRs are activated) (Tables 1, S2 and S3). Some of this effect might be explained by the number of DUX4-binding sites within each peak: 65% of MLT2A1 elements contain more than one good DUX4-motif, compared to only 9% of a class of elements that are less frequently activated, the THE1B-MaLRs (1.0% bound THE1Bs are activated).

### At least 180 MaLR and ERV internal regions are activated by DUX4

To explore the biological significance of DUX4's ability to bind and activate various repetitive elements, we used RNA-seq data to examine the types of transcripts that result from repeat activation. First, we asked whether full-length repetitive elements are activated. 100,864 regions of the human genome assembly are annotated as internal regions of ERV or MaLR elements, or fragments thereof, and many of these repetitive elements are old enough to have acquired sequence changes that allow unique mapping of short sequence reads. RNA-seq data from *DUX4*-transduced myoblasts shows that 184 of these regions are activated in the presence of DUX4 (≥2-fold, FDR-adjusted p-value≤0.1), of which 120 are MaLRs and the rest a mix of other ERV classes. These activated internal MaLR/ERV regions tend to be flanked on one or both sides by a DUX4-bound LTR, where transcription seems to initiate. Repeats whose internal regions are activated tend to be younger than repeats that do not show obvious activation, considering only repeats flanked by at least one DUX4-bound LTR (Figure S2). It is tempting to suggest that this finding indicates that repeats have been evolving towards better DUX4 response, but might more likely reflect the fact that, because less evolutionary time has elapsed, younger repeats have retained sequence elements needed to produce stable transcripts, like TATA boxes and polyadenylation signals.

Using RT-PCR, we were able to verify DUX4-mediated activation of a THE1C-MaLR element and one ERVL element, but not of a second ERVL element (Table 2, Figures S3, S4 and S5). Our inability to confirm activation of one of the ERVLs could be explained if mismapping of RNA-seq reads among highly related ERVL elements misled our choice of an individual element from which to design primers for this assay.

**Table 2:** RT-PCR confirms novel transcripts and shows their presence in testis and FHSD patient cells.

	Myoblasts, lenti-*DUX4*	Myoblasts, lenti-*GFP*	FSHD2 myotubes	Control myotubes	Testis	Liver	Heart	Cerebellum	Kidney	Placenta
THE1B-*HEY1*	+^1^	−	+	−	+	+	+	+	+	+
MLT1B-*PPCS*	+	−	+	−	+	−	−	−	−	−
MLT1E1A-*NT5C1B*	+	−	−	−	+	−	−	+	−	−
MLT1C-lncRNA	+	−	+	−	+	+	−	+	+	+
THE1C-lncRNA	+	−	−	−	+	−	−	(+)	−	−
MLT1D-*DDX10* antisense	+	−	+	−	+	−	−	−	−	−
THE1C internal region	+	−	+	−	+	+	+	−	+	+
ERVL internal region (chr14)	+	−	+	−	+	−	−	−	−	−
ERVL internal region (chr11)	−	−	+	−	+	+	−	(+)	−	(+)

1 “+” symbols indicate that an RT-PCR product was obtained and verified by Sanger sequencing.

“−” symbols indicate samples that were either negative, or gave only bands derived from unrelated loci (our primers recognize repetitive elements, so clean amplification is sometimes difficult).

“(+)” symbols indicate that an RT-PCR product of the expected size was obtained, but could not be cloned for sequencing.

No products were obtained from corresponding negative control samples prepared without reverse transcriptase.

Of these 184 transcriptionally activated ERV or MaLR internal elements, only one contains an ORF exceeding 300 amino acids in length. This full-length THE1D-MaLR internal region aligns to the THE1 consensus sequence without stops or frameshifts, and encodes a 464 amino acid ORF. Because the function of MaLR-encoded proteins is unknown, it is difficult to interpret this finding, but we note that this chromosome 7 THE1 is the only THE1 element in the genome that encodes an uninterrupted ORF and thus is a candidate “active” MaLR element. To examine the possibility that this ORF might be preserved as a domesticated protein, we collected its sequence from other primate genomes. We found that although the ORF is also maintained in chimpanzee and gorilla, it is interrupted by stop codons and/or frameshifts in orangutan, macaque, baboon, and marmoset. The lack of conservation among primates suggests that the ORF does not confer selective advantage. 17 other internal regions contain one or more ORFs exceeding 200 amino acids (arbitrary threshold) and encode fragments of various THE1 internal ORFs as well as fragments of some ERV gag and pol ORFs, although none encode full-length proteins.

### DUX4-bound repetitive elements form alternative promoters for human genes

**Figure 4:**
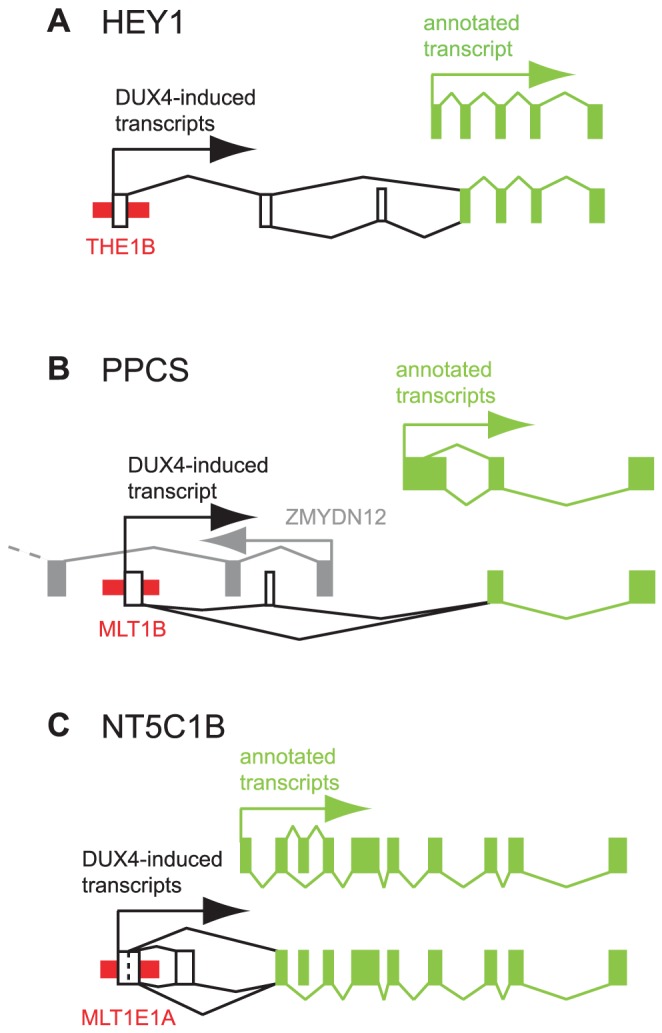
Examples of DUX4-bound repeats that function as alternative promoters for annotated genes. In each panel, thin red boxes depict DUX4-bound repetitive elements. Exons of previously annotated transcripts are depicted as green boxes, with alternative DUX4-induced exons as empty boxes (not to scale). Arrows show the direction of transcription. Diagonal lines show splicing, with alternative splice forms shown above and below the exons. (A) *HEY1*. (B) *PPCS*. An upstream, divergently transcribed gene, *ZMYDN12*, is shown in gray. (C) *NT5C1B*.

Repetitive elements, especially LTRs, can be co-opted as alternative promoters for mammalian genes [32], [33] and can rewire transcriptional networks during evolution [27], [28], [31]. Therefore, acquisition of the *DUX4* retrogene and the spread of repetitive elements around the genome had the potential to alter germ cell promoter usage and the germ cell transcriptional network during primate evolution. Analysis of spliced RNA-seq reads that join a DUX4-bound region with a gene sequence identified 238 previously unannotated DUX4-activated promoters for human genes (Table S4) with 144 of those promoters in repetitive elements. Neither GO nor GREAT analyses [39] of these genes and regions revealed striking enrichment of particular functional classes, although we note that germ cell genes (especially primate-specific genes) are likely very poorly annotated. We selected three genes (*HEY1*, *PPCS* and *NT5C1B*) for validation (Figures 4, S6, S7 and S8, Table 2).

*HEY1* (hairy/enhancer-of-split related with YRPW motif 1) inhibits myogenesis by repressing myogenin and Mef2C [40], and its ∼13-fold activation by DUX4 in muscle cells could inhibit muscle differentiation in FSHD. RNA-seq data from *DUX4*-expressing myoblasts suggests the existence of transcripts that initiate ∼40 kb upstream of *HEY1*'s first annotated exon in a THE1B-MaLR retrotransposon and splice via two additional exons to the second exon of *HEY1* (Figure 4A). These chimeric transcripts encode an ORF with an in-frame start codon in exon 2 that lacks the first 38 amino acids of the full-length HEY1 protein. We verified this THE1B-*HEY1* fusion transcript by RT-PCR, 5′-RACE and Sanger sequencing and showed that its presence in myoblasts is *DUX4*-dependent (Table 2, Dataset S1). This THE1B element is present at the syntenic location in Old and New World monkeys but not in more distant genomes, demonstrating that it inserted in our genome ∼40–75 million years ago [41], long after the origin of the *HEY1* gene.

A DUX4-bound MLT1B-MaLR element initiates *DUX4*-dependent transcripts ∼6 kb upstream of the phosphopantothenoylcysteine synthetase gene (*PPCS*) that splice to exon 2 of *PPCS*, as suggested by RNA-seq data and verified by RT-PCR, 5′-RACE and Sanger sequencing (Figure 4B, Table 2, Dataset S1). The predicted translation start codon of the chimeric transcript in exon 2 is also used in the shorter of the two annotated *PPCS* isoforms (RefSeq NP_001070915). This MLT1B element is found in the syntenic location in diverse mammalian genomes including those of primates, rodents, carnivores and bats, indicating that it inserted in our genome at least 90 million years ago.

A DUX4-bound MLT1E1A-MaLR element initiates transcripts ∼6 kb upstream of the *NT5C1B* (5′-nucleotidase, cytosolic IB) gene and uses either of two donor sites to splice to exon 2 of *NT5C1B* (Figure 4C). This fusion transcript encodes either an ORF lacking the first 14 amino acids of NT5C1B, or a chimeric ORF with the first 10 amino acids of NT5C1B replaced by 17 amino acids encoded in the MLTE1A sequence. Our RNA-seq data shows that the gene is transcribed at low levels in control myoblasts, but is induced ∼300-fold in the presence of DUX4; RT-PCR, 5′-RACE and Sanger sequencing confirm the novel MLT1E1A-*NT5C1B* fusion transcript (Table 2, Dataset S1). This MLT1E1A element is found at the syntenic location in diverse placental mammalian genomes, so must have inserted into the ancestral genome at least 98 million years ago.

### Repetitive elements comprise promoters for lncRNAs and antisense transcripts

**Figure 5:**
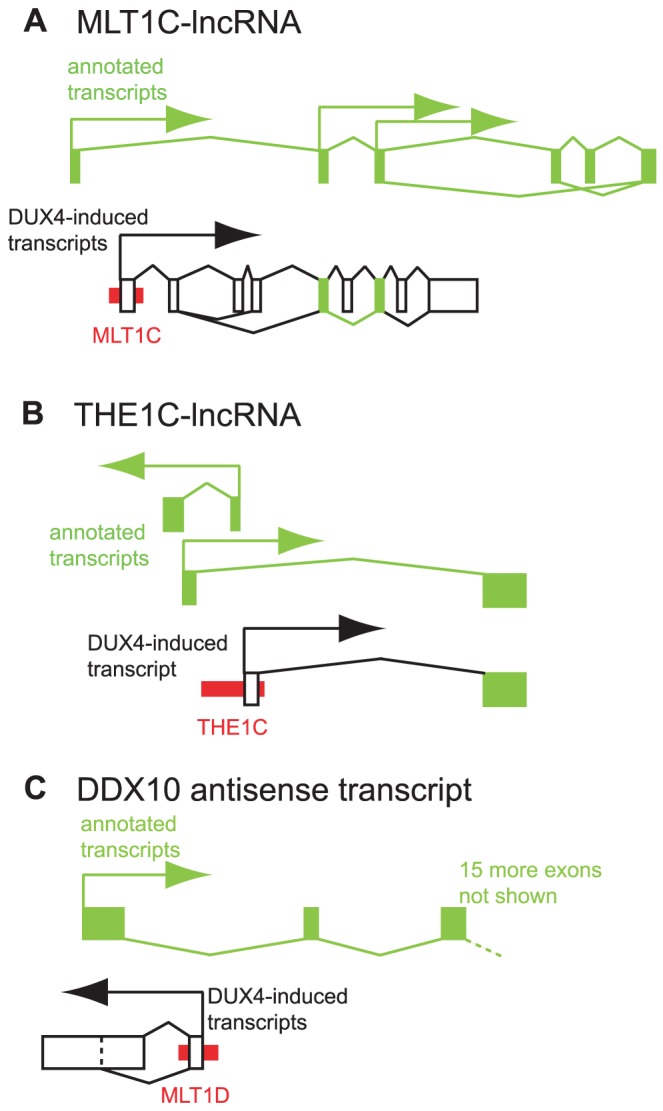
Examples of DUX4-bound repeats that function as alternative promoters for lncRNAs or antisense transcripts. In each panel, thin red boxes depict DUX4-bound repetitive elements. As in Figure 4, exons of previously annotated transcripts are depicted as green boxes, with alternative DUX4-induced exons as empty boxes (not to scale). Arrows show the direction of transcription. Diagonal lines show splicing, with alternative splice forms shown above and below the exons. (A) A lncRNA initiated at an MLT1C element. Two exons of the DUX4-activated lncRNA overlap with exons of previously described lncRNAs TCONS_00003193, TCONS_00002742, TCONS_00002660 and TCONS_00003194 [42]. (B) A lncRNA initiated at an THE1C element that shares the second exon of lncRNA TCONS_00022347 [42]. Additional lncRNAs on the opposite strand also initiate in this region - for clarity, only one is depicted here. (C) An antisense RNA initiated at a DUX4-bound MLT1D element that overlaps the first exon of *DDX10*.

In addition to creating novel first exons for protein-coding genes, our RNA-seq data revealed that DUX4-bound repeats can also create promoters for long non-coding RNAs (lncRNAs). Comparison of DUX4 binding and activated transcripts to a recently published dataset of lncRNAs [42] shows that 18 DUX4-bound sites initiate transcripts for lncRNAs, of which 13 are in repetitive elements (Table S5). We used RT-PCR and Sanger sequencing to verify two of these activated lncRNAs; one initiates in an MLT1C-MaLR element shared among many mammals, and one in a primate-specific THE1C-MaLR element (Figures 5A, 5B, S9, S10). lncRNA catalogs are incomplete and more instances of DUX4-initiated lncRNAs are likely to exist. Two very recent reports [43], [44] show that repetitive elements are enriched at the transcription start sites of lncRNAs. Only 56 of the 2045 repeat-initiated lncRNAs (2.7%) described in one of those reports [43] start in DUX4-bound repeats. We note that the catalogs of lncRNAs used in these two recent studies include transcripts expressed in a diverse set of tissues; if suitable data are available in future, it will be interesting to determine whether DUX4-bound repeats comprise a greater proportion of lncRNA transcription start sites in germ cells than in other tissues.

DUX4-bound sites also initiate transcripts antisense to annotated genes. For example, a transcript that initiates in an MLT1D-MaLR element overlaps the first exon of the *DDX10* gene in the antisense orientation (Figures 5C, S11), and is confirmed by RT-PCR and Sanger sequencing. We are not aware of any genome-wide catalog of antisense transcripts and thus did not perform a systematic analysis of these RNAs.

### Copies of the pericentromeric satellite HSATII are massively activated by DUX4

**Figure 6:**
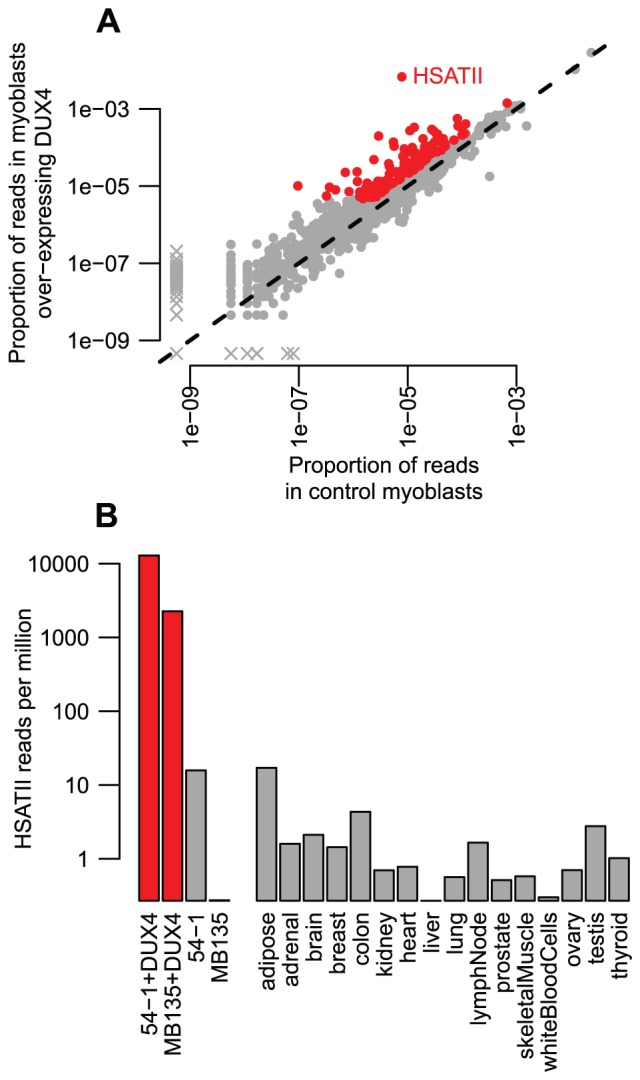
Read-based enrichment analysis of RNA-seq data shows activation of several repeat types. (A) For each repeat type, we show the proportion of RNA-seq reads in two myoblast cell lines transduced with lentiviral *DUX4* (y-axis) compared to the proportion from the same two myoblast cell lines without *DUX4* transduction (x-axis). Repeats plotted in red show ≥2-fold activation and ≥1000 reads summed across the two *DUX4*-expressing myoblast lines. The HSATII repeat is labeled. As in Figure 1A, to ensure all repeat types are shown on this log-scaled plot, zero values are replaced with an arbitrarily low value and points shown with an “x”. (B) HSATII is massively activated by DUX4. For our four RNA-seq samples, and sixteen “Body Map” tissues sequenced by Illumina, we show the proportion of reads that match HSATII pericentromeric satellite repeats on a log10-scale. *DUX4*-expressing cell lines are shown in red, and have a much higher level of HSATII expression than any other sample surveyed.

The analysis above relies on RNA-seq reads that map to fewer than 20 genomic locations and is blind to highly repeated sequences. Therefore, we also used an alternative read-based method to calculate repeat enrichment among DUX4-activated transcripts (see above, and Methods). We identified many of the same repeat classes already highlighted by our analyses of uniquely mappable reads, indicating that many DUX4-bound and activated repeats have diverged enough that standard methods are effective (Figure 6A, Tables S2 and S3). However, as with ChIP-seq data, the read-based analysis uncovered additional classes of activated repeats that were not obviously enriched when we used uniquely mapped reads, including a number of LTR elements, mostly of the ERV1 and ERVK families (e.g. MER52D, LTR12D, MER50B) (Table 1, S2 and S3). Most notably, however, copies of the pericentromeric satellite repeat HSATII are massively activated in the presence of DUX4. Combining the two *DUX4*-transduced myoblast RNA-seq datasets, HSATIIs are activated ∼860-fold with ∼6700 reads per million in *DUX4*-expressing cells compared to only ∼8 reads per million in control samples (Figure 6B), a baseline consistent with low HSATII expression (0.2–17 HSATII sequences per million reads; median 0.9 reads per million) in a panel of sixteen normal tissues sequenced by Illumina (the “Body Map 2.0” dataset, GEO accession GSE30611). We aligned HSATII RNA-seq reads to the HSATII consensus sequence, finding that multiple variant sequences (and therefore multiple repeat units) are transcribed (Figure S12).

Our ChIP-seq data also demonstrated that HSATIIs are bound by DUX4, with ∼1.9-fold enrichment of HSATII sequences among individual reads and 30 DUX4 peaks in mappable HSATII regions (∼5-fold enrichment). Furthermore, each HSATII ChIP-seq peak appears to derive from multiple tandemly-arranged DUX4 binding sites. The 30 HSATII peaks span bigger genomic regions (median peak width 1.2 kb) than other ChIP-seq peaks (median width 0.4 kb) and contain multiple matches to DUX4's consensus motif - the 299 annotated HSATII regions in the human genome assembly contain a total of 820 DUX4 motifs.

### DUX4-bound repeats are promoters in testis and FSHD patient myotubes

We show above that a large number of repeat-initiated transcripts are induced in myoblasts over-expressing *DUX4*. To determine whether these transcripts are expressed in normal germ cell biology and in FSHD muscle, we used RT-PCR to assay for their presence in FSHD patient cells and various tissues from healthy individuals, using Sanger sequencing to confirm that each amplified product derives from the expected locus (Table 2). We found that most of the repeat-initiated transcripts we tested are expressed in myotube cells derived from an FSHD2 patient where disease-causing mutations result in de-repression of endogenous *DUX4*, whereas we did not observe these transcripts in control myotubes that do not express *DUX4*. This indicates that the low level of endogenous DUX4 present in FSHD muscle cells is sufficient to transcriptionally activate the same endogenous repetitive elements identified by our over-expression studies described above.

Given the normal expression of *DUX4* in testis, we assayed these repeat-initiated transcripts in human testis RNA from an individual unaffected by FSHD. We found that all tested repeat-initiated transcripts that respond to DUX4 in skeletal muscle are normally expressed in testis (Table 2), demonstrating that DUX4-repeat binding likely regulates transcription in the male germline.

It is possible that factors other than DUX4 might also regulate transcription from these repetitive elements, perhaps explaining why we also detected some of these transcripts in other normal somatic tissue samples where *DUX4* is not expressed (Table 2). For example, the internal regions of some ERVL and THE1C full-length repeats appear expressed in many tissues – we note that our primers recognize many copies of these repeats, and expression of only a single copy would enable us to detect transcription. Further research is needed to determine whether other transcription factors bind repetitive element promoters in those tissues. Other *DUX* family members might fill this role; their expression patterns and binding specificities are currently unknown.

**Figure 7:**
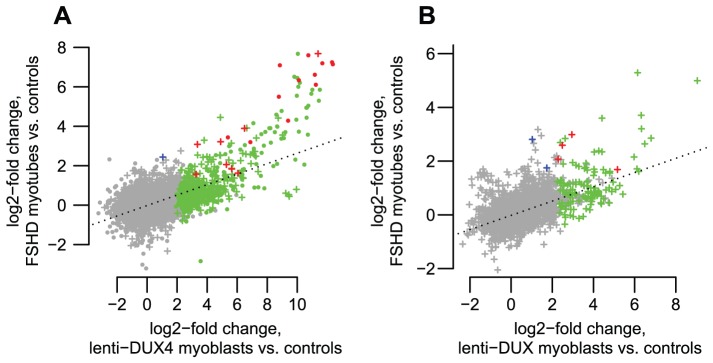
DUX4-bound regions are similarly activated in FSHD patient myotubes and in *DUX4*-transduced myoblasts. (A) DUX4-bound regions show correlated activation levels in FSHD patient myotubes and in our *DUX4*-transduced myoblast experimental system. We show log2-activation levels in each system, counting RNA-seq reads within an arbitrary 1 kb of DUX4-bound regions as for Figure 3A. “+” symbols show bound repetitive elements and dot symbols are unique regions. Green symbols show regions that reach statistical significance in only the *DUX4*-transduced myoblasts, blue symbols are significant in only the FSHD myotubes, and red symbols are significant in both comparisons. The dotted line is a regression line (slope 0.265). (B) Internal regions of ERV and MaLR repeats show correlated activation levels in FSHD patient myotubes and in our *DUX4*-transduced myoblast experimental system. Colors as in panel A. The dotted line is a regression line (slope 0.270).

To further assess transcripts of repetitive elements in FSHD muscle cells, we performed a focused analysis of a small dataset of RNA-seq data from myotubes cultured from three control muscle and two FSHD1 muscle biopsies (Yao *et al.*, manuscript in preparation). RNA-seq profiles show that most of the genes, lncRNAs and internal repeat regions we tested using RT-PCR are expressed in FSHD myotubes but not controls (Figures S3, S4, S5, S6, S7, S8, S9, S10, S11). In regions within 1 kb of DUX4-bound sites (both bound repetitive elements and unique sites), ratios of expression in FSHD myotubes versus controls are well-correlated with the activation levels we found in lenti-*DUX4* transduced myoblasts (Figure 7A, Spearman's rho = 0.38, p<10^−15^). Expression ratios of internal MaLR/ERV regions in FSHD myotubes versus controls are also well-correlated with activation levels in lenti-*DUX4*-transduced myoblasts (Spearman's rho = 0.49, p<10^−15^, Figure 7B). In addition, 13% of the 144 DUX4-bound repetitive elements that form alternative promoters for annotated genes show FSHD-specific transcripts and HSATII is expressed at ∼26-fold higher levels in FSHD myotubes (median ∼2.2 reads per million) than control myotubes (median 0.08 reads per million). Therefore, the endogenous *DUX4* that is expressed in just a subset of FSHD muscle cells is sufficient to drive expression from bound repetitive elements. We also performed a similar focused analysis using testis RNA-seq from the Illumina BodyMap data but the expression level of *DUX4* was very low. Only the most abundant DUX4 targets (according to our *DUX4*-transduced myoblast data) were detected in the testis RNA-seq despite our ability to detect all tested transcripts by RT-PCR (see Table 2) most likely because only a small proportion of cells in the testis express *DUX4* [9].

## Discussion

In this study we show that DUX4 binds many LTR repetitive elements of the MaLR and ERV families and initiates transcription at a number of those elements. Some DUX4-bound LTRs produce retrotransposon transcripts and others form previously unrecognized alternative promoters for human protein-coding genes, lncRNAs, and antisense transcripts. DUX4 also binds and activates transcription of the pericentromeric satellite HSATII. We initially identified these DUX4-activated transcripts in myoblasts transduced with lentivirally-expressed *DUX4*, but show that many of the same loci are transcribed in FSHD but not control muscle cells, indicating that endogenously expressed *DUX4* can activate LTR-driven transcription in FSHD muscle. We also show that all loci we tested using RT-PCR are expressed in the testis of an unaffected individual, suggesting that DUX4 drives transcription from at least some repetitive elements during normal development.

Transposable elements can generate evolutionary novelty by exaptation [28], [45]: their protein-coding regions can evolve to form a host gene, for example the mammalian placentation gene syncytin [46], or their regulatory elements can affect the expression patterns or post-transcriptional control of pre-existing host genes [27], [28]. Barbara McClintock initially proposed that transposable elements could alter expression of neighboring genes [47], and her hypothesis is now supported by a growing body of literature describing repetitive elements that regulate transcription of host genes [32], [33]. In some cases, repetitive elements of a particular family are enriched upstream of genes in similar functional classes [27], [31], [34] and may have provided a means to rewire transcriptional networks, distributing new transcription factor binding sites around the genome in short evolutionary time. We find that DUX4-bound MaLR and ERV repeats are used as alternative promoters for host genes and, at least in some cases, modulate gene expression in human testis. Although other TFs have been shown to bind LTR elements [29]–[31], we provide the first demonstration of a transcription factor that binds and activates the MaLR subfamily of LTR elements, and a possible explanation for the rapid evolution of testis expression patterns that has been observed in mammals [35]. In a possible parallel with our results, Peaston *et al.* found that MaLR elements initiate dozens of genic transcripts in mouse oocytes [48], although they did not identify the transcription factor(s) involved.

DUX4-induced repeat-initiated transcripts also include a number of lncRNAs. Although the human genome contains several thousand lncRNAs, functions have been determined for only a few. Even those few functions appear diverse, including recruitment of chromatin-modifying factors, involvement in enhancer function, organization of nuclear substructures, and control of translation [49], [50]. Many lncRNAs are testis-specific [42], raising the question of whether DUX4 might be responsible for transcription of a subset of lncRNAs in the testis. In the future when lncRNA catalogs are more complete and their functions have begun to be elucidated, it will be interesting to revisit the question of whether DUX4-bound repeats played a role in the evolution of the germline lncRNA transcriptional network.

DUX4 also activates the transcription of relatively intact copies of ERV and MaLR retrotransposons that do not splice to genes or lncRNAs, bringing up the possibility that it had a role in their genomic spread. In order for a retroelement to successfully invade the mammalian genome, it must be active in germ cells. However, because retrotransposition can also be harmful to the host organism [22], [23], retroelements whose activity is strictly restricted to germ cells could have an evolutionary advantage. The germline transcription factors that activated MaLR and ERV retrotransposition are currently unknown: the expression of *DUX4* in testis and repression in other tissues together with its ability to bind and activate MaLR and ERV elements could suggest it had a role in repeat mobilization during evolution.

DUX4's activation of relatively intact ERV and MaLR copies might additionally suggest that it currently has a role in their developmental epigenetic silencing. Recent years have yielded an increasing understanding of the mechanisms eukaryotes employ to defend against repetitive elements, including piRNA pathways and mechanisms that establish repressive chromatin marks [51], [52]. These crucial defenses against repetitive elements are particularly active in germ cells and the early embryo and require an initial transcriptional activation of the retrotransposon to feed into the “ping-pong” cycle that produces and amplifies piRNAs that then silence homologous sequences [51]. It would be interesting in future to investigate whether DUX4 is involved in the initial activation of retrotransposon transcription in germ cells. Other pathways also exist to silence repetitive elements. For example, KAP1 controls endogenous retroviral elements by recruiting chromatin-modifying factors [53], [54]; it is targeted to murine leukemia virus LTRs in a sequence-specific fashion by the KRAB-zinc finger protein ZFP809 [55]. To explain KAP1's more general role in ERV silencing, it is assumed that other sequence-specific DNA-binding proteins exist to target it to other ERV classes. The large number of diverse zinc finger proteins present in the mouse and human genomes may fill this role [56], but the sequence-specificity of DUX4 for LTRs might also allow it to recruit repressive factors to repetitive elements in a cell-type specific context.

Similar to retroelements, the pericentromeric satellite HSATII is bound and activated by DUX4. HSATII is a pericentromeric satellite sequence, repeated in large tandem arrays close to a subset of human centromeres. Its consensus sequence is 170 bp long [24] and comprises ∼6 imperfect tandem copies of a smaller ∼25–28 bp repeat unit (data not shown). Pericentromeric regions show evidence of transcriptional activity during specific stages of male meiosis [57], and transcription of satellite sequences at early developmental stages appears be an important prerequisite for later establishment of repressive heterochromatin [58]. The activation of both interspersed repetitive elements and HSATII by DUX4 and its expression in germ cells of the testis could suggest a role in establishing repressive heterochromatin at both dispersed transposons and in tandemly repeated sequences near centromeres. Transcription of repetitive elements and satellite sequences in other, less appropriate biological contexts would likely be harmful, perhaps giving a strong evolutionary advantage to the location of the *DUX4* retrogene within a high copy-number macrosatellite that can be tightly repressed by similar epigenetic means.

*DUX4* is normally expressed in the testis and epigenetically repressed in somatic tissues, but its variegated de-repression in muscle cells causes facioscapulohumeral muscular dystrophy (FSHD) [9], [13]–[15]. Previous work has provided some insight into why *DUX4* over-expression is pathogenic [10], [59]–[61]. The repeat-initiated transcripts we describe here could also contribute to FSHD pathogenesis. For example, the *HEY1* gene (induced by DUX4-mediated activation of an upstream THE1B element) can inhibit myogenesis by repressing myogenin and Mef2C [40], and its activation might contribute to the muscle deficiencies seen in FSHD. In addition, expression of satellite transcripts can cause genomic instability [62], [63]; DUX4-mediated activation of HSATII might similarly affect FSHD muscle cells. DUX4-induced expression of ERV and MaLR-encoded proteins or protein fragments could also have functional consequences in testis or FSHD muscle cells. Notably, some ERV-encoded env proteins contain a peptide with immunosuppressive properties [64], perhaps contributing to the suppression of innate immunity we observe upon *DUX4* over-expression in myoblasts [10]. Conversely, ERV-encoded protein fragments could be antigenic, and might elicit an immune response and some of the inflammation seen in FSHD muscle [65], [66].

Our findings may also have clinical implications for cancer biology. The DUX4 target HSATII is expressed in a number of cancers [67], and it has been shown that mouse cells lacking the genome caretaker gene *Brca1* aberrantly transcribe satellite sequences leading to genome instability [62]. A large number of other DUX4 targets are known “cancer testis antigens” (CTAs): genes normally expressed only in testis but de-repressed in some cancers, eliciting an immune response [10]. Furthermore, in Hodgkin's lymphoma cells, a THE1B-MaLR element provides an alternative promoter for the *CSF1R* proto-oncogene and de-repression of THE1B elements is widespread [68]. Together with the previous finding that the *DUX4*-containing D4Z4 repeat is hypomethylated in certain tumors [69], these observations raise the question of whether *DUX4* de-repression in cancers might mediate the activation of HSATII, CTAs and/or THE1B promoters.

*DUX4* is a primate-specific retrogene and a member of a small gene family that has experienced substantial change during mammalian evolution [1]–[3]. Although the binding preferences and functions of primate *DUX4* orthologs and of *DUX* paralogs are still unknown, we note that an alignment of *DUX* family homeodomain sequences [1] shows that at least some of the residues predicted to determine DNA-recognition preferences [70] are different between *DUX4* and the parental *DUXC* gene. Determining whether other members of the *DUX* gene family also bind and regulate retrotransposons will illuminate the importance of these largely unstudied genes and retroelements in the biology and evolution of mammalian germ cells and in muscle disease.

## Materials and Methods

### General methods and genome-wide datasets

All experiments were performed with approval of the Institutional Review Board of the Fred Hutchinson Cancer Research Center.

We wrote a number of custom scripts using R [71], PERL, and several Bioconductor [72], [73] and Bioperl functions [74]. We use the hg19 (GRCh37/February 2009) reference human genome assembly and annotations provided by UCSC Genome Bioinformatics [75], including the RefSeq [76], lncRNA [42] and common SNP tracks, and phyloP scores for primates and placental mammals [77]. We also used RepeatMasker [78] analysis of the human genome assembly obtained via the UCSC site (chromOut.tar.gz, which uses “RELEASE 20090120” of RepBase [24]). This version of RepBase recognizes ∼1400 human repetitive element types, classified into 56 families, with families classified into 21 classes. We obtained repeat consensus sequences from RepBase [24]. We used the Bioconductor GOstats package [79] to test for GO term enrichment, and GREAT analysis [39] was performed online (http://great.stanford.edu).

### Estimation of repeat enrichment among ChIP-seq peaks

Our ChIP-seq data were previously published [10] (GEO accession GSE33838). Briefly, these 40 bp ChIP-seq reads derive from chromatin immunoprecipitated with a mix of two DUX4 antibodies. Chromatin was extracted from myoblasts transduced with lentivirus carrying *DUX4*, or from negative control myoblasts that do not express *DUX4*. The human genome contains multiple near-identical copies of *DUX4*: in our experiments, we used the full-length splice form of the most distal *DUX4* copy on chromosome 4q35, because this is the copy that appears to be expressed and pathogenic in FSHD patient muscle [9], [10], [13]. This *DUX4* isoform is also expressed in testis, along with other copies containing minor sequence variants whose functional consequences are currently unknown [9]. We mapped each ChIP-seq read to the human reference assembly (hg19) using BWA [80]. We eliminated multiply-mapping reads (retaining reads with mapq >15) and determined peak locations [10]. We identified a position weight matrix (PWM) describing a motif that is strongly enriched among DUX4 peaks, using only the ∼24,000 peaks that do not overlap a repetitive element [10]. We determined a score threshold for this PWM of 9.75, above which >97% of ChIP-seq peaks contain at least one motif.

For further analysis of DUX4 binding sites we refined peak locations by identifying the single 17-mer subsequence with highest score to the DUX4 PWM, rather than using the entire peak region (ChIP-seq resolution is limited by fragment size of ∼200 bp), making the simplifying assumption that each peak contains a single DUX4-binding site. We then determined whether each peak's best-scoring subsequence overlaps a repetitive element. To estimate peak-level enrichment for each repeat type, we divided the proportion of all peaks that overlap each repeat type (observed) by the proportion of base-pairs in the sequenced genome within that repeat type (expected). We previously found that DUX4 binding sites are distributed relatively uniformly across different types of genomic regions (promoters, intergenic regions, introns, etc.) [10], so it is not necessary to adjust our “expected” proportions for the different prevalence of various repeats in these region types.

The DUX4 binding motif includes an average of 5.03 G or C residues among its 17 bases. In order to create an AT-matched set of genomic locations, we randomly selected 17-bp regions from the human genome, eliminating any that overlap assembly gaps, retaining those whose sequence contains 4–6 G or C residues, and downsampling the final set to contain 63,795 sites to match the dataset of DUX4 binding sites. We then determined whether these randomly selected 17-mers overlapped repetitive elements, and counted the types of repeats found among overlapping elements. We performed this sampling 10 times, and use the mean fraction of sites overlapping repeats to calculate enrichment measures shown in the columns of Tables S2 and S3 labeled “peak-based ChIP-seq enrichment estimate, compared to randomly sampled AT-matched regions”.

In order to determine whether DUX4-bound repeats also tend to be bound by other regulatory factors, we obtained ENCODE ChIP-seq peak locations for 161 regulatory factors via the UCSC Genome Bioinformatics “wgEncodeAwgTfbsUniform” tables. For each factor, we chose a single representative ENCODE dataset, and determined the number of peaks that overlap DUX4 ChIP-seq peaks assigned as bound repeats (see above). Using the entire peak regions (several hundred base-pairs wide) for both DUX4 and the other TFs (rather than binding sites defined at higher resolution) allows us to ask the biologically relevant question of whether TFs bind in the vicinity of DUX4, rather than asking whether binding sites are exactly overlapping.

### Estimation of repeat enrichment among ChIP-seq reads

In our standard ChIP-seq analysis (above), we ignored sequencing reads that map to multiple genomic locations to ensure that called peaks likely represent true binding sites. However, this method is blind to binding in very recently duplicated regions, so we used an alternative bioinformatic method very similar to that of Day *et al.* [37]. This method examines repeat enrichment among individual sequencing reads, comparing counts of reads matching each repeat type in a ChIP-seq sample to counts in a negative control sample.

In more detail, we first filtered ChIP-seq read datasets to remove low quality sequences, and reads that match our lentivirus-*DUX4* constructs, the packaging constructs used during lentivirus preparation, or Illumina adapter sequences. We constructed an alternative repeat-based “reference genome”, where each repeat type is represented by a “chromosome” comprising every genomic instance of that repeat, with an amount of flanking sequence on each side equal to half the length of a sequence read, concatenated together with a intervening stretches of Ns that are each longer than a sequencing read. We then used BWA to map filtered reads to the repeat-based reference genomes, without filtering results for uniquely mapping sequences. We used the samtools idxstats program [81] to determine the proportion of filtered reads mapping to each repeat type. We estimated enrichment by comparing the proportion of reads mapping to each repeat in the ChIP sample with the proportion in the control sample, adding 0.5 to each count to avoid problems that would arise from division by zero.

These enrichment estimates are “dampened” because ChIP samples contain background DNA derived from unbound genomic regions (50–90% of reads); although immunoprecipitation depletes unbound sequences it cannot completely eliminate them. Background proportions differ between experimental and control samples, and background fraction undoubtedly contains many repetitive sequences. These read-based estimates are therefore likely a very conservative measure of true enrichment in the bound DNA fraction.

### Transcriptome data

Our RNA-seq data are available from GEO with accessions GSE45883 and GSE51041.

Two human myoblast cell lines (54-1 and MB135) were each transduced with lentivirus carrying *DUX4*. After 48 hours (54-1 cells) or 24 hours (MB135 cells), RNA was extracted, poly(A) selected, and subjected to Illumina sequencing using standard protocols to generate 100 bp single-end reads. As negative controls, we also sequenced RNA from untransduced 54-1 cells, and from MB135 cells transduced with lentivirus carrying *GFP*.

In addition, we sequenced RNA from two FSHD1 and three control myotube samples. Primary myoblast cell lines were received from the University of Rochester biorepository (http://www.urmc.rochester.edu/fields-center) and were cultured in DMEM/F-10 media (Gibco) in the presence of 20% heat-inactivated fetal bovine serum (Gibco), 1% penicillin/streptomycin (Gibco). Media was supplemented with 10 ng/ml rhFGF (Promega) and 1 µM dexamethasone (SIGMA). Myoblasts were fused at 80% confluence in DMEM/F-12 Glutamax media containing 2% KnockOut serum replacement formulation (Gibco) for 36 hours. RNA was extracted, poly(A) selected, and subjected to Illumina sequencing using standard protocols to generate 100 bp single-end reads.

Our analyses are conservative, identifying only the elements with greatest activation extents, because we lack statistical power due to small numbers of samples and a minor technical issue with the 54-1 control sample (see below). In addition, we note that we are only examining polyadenylated transcripts and may be ignoring others; however, full-length transcripts of many repetitive elements are polyadenylated, including those of ERVs, L1s and Alu elements [23], [82].

Our analysis of RNA-seq reads falls into two general categories, both described in detail below.

First, we performed a read-based analysis as we had done for ChIP-seq reads, combining read counts across all instances of a particular repeat type; this method does not allow us to determine which instance of a repeat type is activated, merely that one or more elements of that class shows activation, but unlike standard methods, it does allow examination of recently duplicated sequences. We used the same read-based method as we did for the ChIP-seq reads (see above) on our RNA-seq reads. Although this method uses the BWA read-mapping tool and will therefore fail to map spliced reads, it does not suffer from a limitation of tophat that it suppresses mappings for reads mapping to many genomic locations.

Second, we considered individual genomic locations (repeat instances, genes, lncRNAs, *etc.*) using tophat and DESeq, a method that limits our ability to examine highly similar multicopy sequences. We mapped reads to the genome using tophat [83], allowing up to 20 map locations for multiply-mapping reads (no map location is reported for reads that map to >20 locations). We counted reads overlapping each region of interest (gene, lncRNA, repeat, *etc.*) using the bedtools coverageBed function [84] with the “split” and “counts” options. We filtered regions to retain only those that had at least 10 mapped reads (summed across the four myoblast datasets). We then used the DESeq Bioconductor package [85] to detect differentially expressed regions. We also repeated these analyses after filtering tophat output to retain only reads that map uniquely to the genome - results were very similar to those we obtained using all map locations (data not shown).

We encountered a minor technical issue: the RNA-seq read dataset for one sample, the untreated 54-1 negative control, has very low levels of contaminating reads from a lentivirus-*DUX4* treated sample. A small number of reads match the lentivirus backbone, the *DUX4* insert, and the lentivirus vector-*DUX4* junction. In addition, we find small numbers of reads for genes (and repeats) that are activated to very high levels in *DUX4*-expressing cells but are “off” in cells that do not express *DUX4*, very consistently at about 1/1000 of the number of reads found in 54-1 cells over-expressing *DUX4*. The most likely explanation is that a small amount of RNA from another sample contaminated the untreated 54-1 cell RNA sample before sequencing. Although this issue only affects genes expressed to very high levels, it causes a technical problem for the DESeq statistical analysis method we used, because contaminating reads for genes expressed to high levels make dispersal estimation difficult (data not shown). This issue contributes to the conservative nature of our conclusions.

In order to examine the age of activated internal repeat regions relative to internal repeat regions that do not show obvious activation (Figure S2), we first applied the following filters to the full dataset of 100,864 regions of the human genome assembly annotated as internal regions of ERV or MaLR elements (these regions were obtained via UCSC's track of RepeatMasker data). We first selected repeats flanked by DUX4-bound LTRs, requiring a ChIP-seq peak within 5 kb of the internal repeat region whose best DUX4 motif is within an LTR-type repeat element. We then filtered the dataset to only retain repeat regions spanning ≥500 bp, because repeat ages estimated from shorter regions are likely unreliable. This filtered dataset contains 10,190 internal repeat regions, including 92 of the 184 activated regions. We use the “milliDiv” statistic reported in UCSC's RepeatMasker track (divergence from consensus sequence, per 1000 sites examined) as a proxy for repeat age (lower divergence = younger), dividing the number by 1000 to present a more intuitive per-site divergence measure.

To analyze diversity of transcribed HSATII repeat units among RNA-seq reads (Figure S12), we first extracted all reads that mapped to any HSATII copy in our alternative repeat-based reference genome (see above, in “Estimation of repeat enrichment among ChIP-seq reads” section). We then re-aligned those reads to a consensus-based HSATII reference sequence, comprising a full copy of the 170 bp consensus sequence from RepBase, concatenated to a second partial copy (bases 1–98), because HSATII is found in the genome in large tandemly repeated blocks, and we wanted to ensure we captured any RNA-seq reads that begin in the end of one repeat unit and continue into the beginning of the next unit. We used blastn [86] to align reads to this consensus HSATII sequence, tolerating mismatches, and used a custom PERL script to convert blastn output to sam format so that we could use the IGV browser [87] to view the resulting large alignment.

### Identification of transcripts that initiate at DUX4-bound repeats and splice to annotated genes or lncRNAs

In order to identify DUX4-bound regions that are used as previously unannotated promoters for genes or lncRNAs, we first used bedtools' intersectBed function [84] on tophat's genomic mappings to filter RNA-seq datasets to retain only reads that overlap DUX4 ChIP-seq peaks (DUX4-bound regions). We additionally filtered reads to retain only those that contain an intron of at least 20 bp and that overlap annotated genes (or lncRNAs). After these filtering steps, we created a table of peak-gene (or peak-lncRNA) pairs, counting the number of reads for each peak-gene pair in each RNA-seq dataset. We eliminated any peak-gene pairs where the peak and the gene themselves overlapped, and further focused on pairs linked by at least one read in both of the *DUX4*-overexpressing myoblast cell lines (or in both of the FSHD patient myotube samples). For each peak-gene (or peak-lncRNA) pair, we estimated a *DUX4* activation ratio, by comparing the proportion of reads linking that peak and gene in the two *DUX4*-expressing myoblasts (or two FSHD patient myotubes) with the proportion of reads in the two control myoblast samples (or three control myotubes). Again, we added 0.5 to each read count to avoid problems with division by zero. We then filtered the peak-gene (or peak-lncRNA) list to only include pairs with a *DUX4* activation ratio of ≥2.

### RNA samples used for RT-PCR and 5′-RACE of selected transcripts

Human tissue RNAs were purchased from BioChain (Hayward, CA) and had been DNase-treated by the supplier.

Primary human myoblasts (54-1 and MB135, neither of which has an FSHD mutation, and MB200, from an individual with FSHD2) were collected and cultured as previously described (Snider et al., 2010). 54-1 primary myoblasts were transduced with a lentiviral vector expressing either *DUX4* or *GFP* (as in our RNA-seq experiments). 24 hours after transduction, RNA was harvested for RT-PCR or 5′ RACE. Non-transduced 54-1 and MB200 cells were differentiated into myotubes by growing to 100% confluency and adding differentiation media for 48 hours (Dulbecco's Modified Eagle Medium, 1% penicillin-streptomycin, 1% horse serum, 0.1% insulin, 0.1% transferrin).

Total RNA was isolated from cultured cells using the RNeasy Mini Kit (Qiagen) followed by Invitrogen's protocol for DNase I (Amplification Grade) treatment with the addition of RNaseOUT (Invitrogen) to the reaction. DNase I reaction components were removed using the RNeasy Mini Kit (Qiagen). RNA was eluted using 50 µl of RNase-free water, and the volume was reduced using a SpeedVac.

### RT-PCR

cDNA synthesis was performed using 1 µg of RNA, SuperScript III reverse transcriptase (Invitrogen) and random hexamers (Roche) according to the manufacturer's instructions (50**°**C 30 min and then 55**°**C 30 min). Reactions were cleaned using the QIAquick (Qiagen) PCR purification system and eluted with 50 µl of water. Negative control samples corresponding to each cDNA sample were prepared by omitting reverse transcriptase.

PCR reactions were performed with 10% PCRx Enhancer solution (Invitrogen) and Platinum Taq polymerase (Invitrogen) using 10% of the cDNA reaction as template in a total reaction volume of 20 µl in thin-walled MicroAmp reaction tubes (Applied Biosystems). Primers are listed in Table S6. PCR cycling conditions for cell culture samples were 95**°**C for 5 min, followed by 35 cycles of 95**°**C for 30 s, 55**°**C for 30 s and 68**°**C for 2 min, followed by a final extension of 7 minutes at 68**°**C. Cycling conditions for human tissue samples were the same, except that 45 cycles were used. PCR products were examined on 1% UltraPure (Invitrogen) agarose gels in TBE, cloned and sequenced using BigDye Terminators (Applied Biosystems).

### 5′ RACE

5′ RACE for the THE1B-*HEY1*, MLT1B-*PPCS*, and MLT1E1A-*NT5C1B* transcripts was performed on total RNA using the GeneRacer kit (Invitrogen). Prior to PCR with gene-specific primers and GeneRacer 5′ primers, the RT reaction was cleaned using QIAquick spin columns (Qiagen) as described above. Gene-specific reverse primers are listed in Table S6. PCR products were gel purified, cloned into TOPO 4.0 (Invitrogen) and sequenced using BigDye Terminators (Applied Biosystems).

## Supporting Information

Dataset S1 5′ RACE sequences. For *HEY1*, *PPCS* and *NT5C1B*, we provide the sequence of the 5′ RACE clone that extends furthest upstream, in fasta format.
(TXT)

Figure S1 Consensus sequences for most bound repeat types contain a DUX4 binding motif. We scanned repeat consensus sequences with a PWM representing DUX4's binding preferences. In all panels, the dashed gray vertical line represents an ad hoc PWM score threshold of 9.75: most ChIP-seq peaks exceed this threshold. (A) For each repeat type, we plot read-based ChIP-seq enrichment estimate (y-axis) against best PWM score in the consensus (x-axis). We show only repeats with ≥1000 mapped reads, because enrichment estimates derived from fewer reads are less biologically significant and more error-prone. Orange and red datapoints show repeats enriched (≥2-fold) among DUX4 binding sites by either the peak-based (≥100 peaks) or the read-based method (≥1000 reads); most contain a good DUX4 motif in their consensus sequence. To explore exceptions, we investigated three repeat types: MLT1A (red square, and panel B); THE1A (red triangle, and panel C);and HAL1 (black diamond, and panel D). (B, C, D) Distribution of best PWM scores for every genomic instance of each repeat type, demonstrating that consensus motif scores may not represent genomic instances well. Blue vertical lines show best consensus motif scores. Text below each title shows how many genomic instances of the repeat contain ≥1 motif scoring ≥9.75. (B) MLT1A repeats are enriched among DUX4 binding sites. Although their consensus has no good motif, a reasonable proportion of genomic repeat instances do, explaining DUX4 binding. (C) Some repeats, like THE1As, have a good motif in their consensus and are enriched for DUX4 binding; many genomic instances contain a motif identical to that of the consensus sequence. (D) Other repeats, like HAL1s, have a good motif in their consensus but only in a few genomic instances, explaining the lack of DUX4 binding. Such discrepancies between consensus PWM scores and repeat instances can occur because repeat instances acquire post-insertion mutations, and/or because of inaccuracy in the consensus.
(PDF)

Figure S2 Activated internal repeats are younger than those that do not show activation. Repeat age is estimated using divergence per site from consensus sequence as a proxy (as reported by RepeatMasker). We show divergence for a filtered dataset of the internal regions of LTR-type elements that are close to DUX4-bound LTRs, showing those that are transcriptionally activated to a statistically significant level (“activated”) separately from the remaining elements (“not activated”). The activated elements show lower divergence from consensus sequences (i.e. are younger) than the remaining elements.
(PDF)

Figure S3 UCSC browser screenshot showing details of THE1C genomic region, including RNA-seq data. We use the UCSC Genome Browser [88] to display locations of repetitive elements and genes, and use custom tracks to show various additional features and as well as data generated in our lab. We created the blue and red tracks labeled “LTRs_showing_type” and “LTR_internal_regions_showing_type” by filtering UCSC's RepeatMasker track so that only LTR-type repeats are shown (blue, only the long terminal repeats; red, only the internal regions), along with a label for each repeat that shows the repeat family and subtype. The pink “DUX4_ChIPseq” track shows fragment coverage in our ChIP-seq experiment, and the “DUX4_ChIPseq_peaks” track shows the 63,795 peaks called from that coverage data. DUX4 motifs show any 17-mer sequence matching the DUX4 PWM with score of ≥9.75 (see Methods), labeled by score. We also include a track showing locations of the primer sequences given in Table S6. The six black tracks with labels ending “_RNAseq” show sequence coverage in our RNA-seq experiments. Each ChIP-seq and RNA-seq coverage track is scaled individually, according to the maximum coverage in that dataset within the viewing window; scales for each track can be seen on the sidebar (e.g. coverage shown for the DUX4_ChIPseq track ranges from 4–70, but ranges from 4–218 for the MB135_DUX4_RNAseq track). The three tracks with labels ending “_RNAseq_splice_junctions” show the number of spliced reads supporting each predicted splice junction, with junctions supported by only 1–10 reads in gray, junctions supported by 11–99 reads in orange, and those supported by ≥100 reads in red.
(PDF)

Figure S4 UCSC browser screenshot showing details of ERVL genomic region on chromosome 14, including RNA-seq data. We use the UCSC Genome Browser [88] to display locations of repetitive elements and genes, and use custom tracks to show various additional features and as well as data generated in our lab. We created the blue and red tracks labeled “LTRs_showing_type” and “LTR_internal_regions_showing_type” by filtering UCSC's RepeatMasker track so that only LTR-type repeats are shown (blue, only the long terminal repeats; red, only the internal regions), along with a label for each repeat that shows the repeat family and subtype. The pink “DUX4_ChIPseq” track shows fragment coverage in our ChIP-seq experiment, and the “DUX4_ChIPseq_peaks” track shows the 63,795 peaks called from that coverage data. DUX4 motifs show any 17-mer sequence matching the DUX4 PWM with score of ≥9.75 (see Methods), labeled by score. We also include a track showing locations of the primer sequences given in Table S6. The six black tracks with labels ending “_RNAseq” show sequence coverage in our RNA-seq experiments. Each ChIP-seq and RNA-seq coverage track is scaled individually, according to the maximum coverage in that dataset within the viewing window; scales for each track can be seen on the sidebar. The three tracks with labels ending “_RNAseq_splice_junctions” show the number of spliced reads supporting each predicted splice junction, with junctions supported by only 1–10 reads in gray, junctions supported by 11–99 reads in orange, and those supported by ≥100 reads in red.
(PDF)

Figure S5 UCSC browser screenshot showing details of ERVL genomic region on chromosome 11, including RNA-seq data. We use the UCSC Genome Browser [88] to display locations of repetitive elements and genes, and use custom tracks to show various additional features and as well as data generated in our lab. We created the blue and red tracks labeled “LTRs_showing_type” and “LTR_internal_regions_showing_type” by filtering UCSC's RepeatMasker track so that only LTR-type repeats are shown (blue, only the long terminal repeats; red, only the internal regions), along with a label for each repeat that shows the repeat family and subtype. The pink “DUX4_ChIPseq” track shows fragment coverage in our ChIP-seq experiment, and the “DUX4_ChIPseq_peaks” track shows the 63,795 peaks called from that coverage data. DUX4 motifs show any 17-mer sequence matching the DUX4 PWM with score of ≥9.75 (see Methods), labeled by score. We also include a track showing locations of the primer sequences given in Table S6. The six black tracks with labels ending “_RNAseq” show sequence coverage in our RNA-seq experiments. Each ChIP-seq and RNA-seq coverage track is scaled individually, according to the maximum coverage in that dataset within the viewing window; scales for each track can be seen on the sidebar. The three tracks with labels ending “_RNAseq_splice_junctions” show the number of spliced reads supporting each predicted splice junction, with junctions supported by only 1–10 reads in gray, junctions supported by 11–99 reads in orange, and those supported by ≥100 reads in red.
(PDF)

Figure S6 UCSC browser screenshot showing details of HEY1 genomic region, including RNA-seq data. We use the UCSC Genome Browser [88] to display locations of repetitive elements and genes, and use custom tracks to show various additional features and as well as data generated in our lab. We created the blue and red tracks labeled “LTRs_showing_type” and “LTR_internal_regions_showing_type” by filtering UCSC's RepeatMasker track so that only LTR-type repeats are shown (blue, only the long terminal repeats; red, only the internal regions), along with a label for each repeat that shows the repeat family and subtype. The pink “DUX4_ChIPseq” track shows fragment coverage in our ChIP-seq experiment, and the “DUX4_ChIPseq_peaks” track shows the 63,795 peaks called from that coverage data. DUX4 motifs show any 17-mer sequence matching the DUX4 PWM with score of ≥9.75 (see Methods), labeled by score. We also include a track showing locations of the primer sequences given in Table S6, and a track that shows sequences of 5′ RACE products. The six black tracks with labels ending “_RNAseq” show sequence coverage in our RNA-seq experiments. Each ChIP-seq and RNA-seq coverage track is scaled individually, according to the maximum coverage in that dataset within the viewing window; scales for each track can be seen on the sidebar. The three tracks with labels ending “_RNAseq_splice_junctions” show the number of spliced reads supporting each predicted splice junction, with junctions supported by only 1–10 reads in gray, junctions supported by 11–99 reads in orange, and those supported by ≥100 reads in red.
(PDF)

Figure S7 UCSC browser screenshot showing details of PPCS genomic region, including RNA-seq data. We use the UCSC Genome Browser [88] to display locations of repetitive elements and genes, and use custom tracks to show various additional features and as well as data generated in our lab. We created the blue and red tracks labeled “LTRs_showing_type” and “LTR_internal_regions_showing_type” by filtering UCSC's RepeatMasker track so that only LTR-type repeats are shown (blue, only the long terminal repeats; red, only the internal regions), along with a label for each repeat that shows the repeat family and subtype. The pink “DUX4_ChIPseq” track shows fragment coverage in our ChIP-seq experiment, and the “DUX4_ChIPseq_peaks” track shows the 63,795 peaks called from that coverage data. DUX4 motifs show any 17-mer sequence matching the DUX4 PWM with score of ≥9.75 (see Methods), labeled by score. We also include a track showing locations of the primer sequences given in Table S6, and a track that shows sequences of 5′ RACE products. The six black tracks with labels ending “_RNAseq” show sequence coverage in our RNA-seq experiments. Each ChIP-seq and RNA-seq coverage track is scaled individually, according to the maximum coverage in that dataset within the viewing window; scales for each track can be seen on the sidebar. The three tracks with labels ending “_RNAseq_splice_junctions” show the number of spliced reads supporting each predicted splice junction, with junctions supported by only 1–10 reads in gray, junctions supported by 11–99 reads in orange, and those supported by ≥100 reads in red.
(PDF)

Figure S8 UCSC browser screenshot showing details of NT5C1B genomic region, including RNA-seq data. We use the UCSC Genome Browser [88] to display locations of repetitive elements and genes, and use custom tracks to show various additional features and as well as data generated in our lab. We created the blue and red tracks labeled “LTRs_showing_type” and “LTR_internal_regions_showing_type” by filtering UCSC's RepeatMasker track so that only LTR-type repeats are shown (blue, only the long terminal repeats; red, only the internal regions), along with a label for each repeat that shows the repeat family and subtype. The pink “DUX4_ChIPseq” track shows fragment coverage in our ChIP-seq experiment, and the “DUX4_ChIPseq_peaks” track shows the 63,795 peaks called from that coverage data. DUX4 motifs show any 17-mer sequence matching the DUX4 PWM with score of ≥9.75 (see Methods), labeled by score. We also include a track showing locations of the primer sequences given in Table S6, and a track that shows sequences of 5′ RACE products. The six black tracks with labels ending “_RNAseq” show sequence coverage in our RNA-seq experiments. Each ChIP-seq and RNA-seq coverage track is scaled individually, according to the maximum coverage in that dataset within the viewing window; scales for each track can be seen on the sidebar. The three tracks with labels ending “_RNAseq_splice_junctions” show the number of spliced reads supporting each predicted splice junction, with junctions supported by only 1–10 reads in gray, junctions supported by 11–99 reads in orange, and those supported by ≥100 reads in red.
(PDF)

Figure S9 UCSC browser screenshot showing details of MLT1C-lncRNA genomic region, including RNA-seq data. We use the UCSC Genome Browser [88] to display locations of repetitive elements and genes, and use custom tracks to show various additional features and as well as data generated in our lab. We created the blue and red tracks labeled “LTRs_showing_type” and “LTR_internal_regions_showing_type” by filtering UCSC's RepeatMasker track so that only LTR-type repeats are shown (blue, only the long terminal repeats; red, only the internal regions), along with a label for each repeat that shows the repeat family and subtype. The pink “DUX4_ChIPseq” track shows fragment coverage in our ChIP-seq experiment, and the “DUX4_ChIPseq_peaks” track shows the 63,795 peaks called from that coverage data. DUX4 motifs show any 17-mer sequence matching the DUX4 PWM with score of ≥9.75 (see Methods), labeled by score. We also include a track showing locations of the primer sequences given in Table S6. The six black tracks with labels ending “_RNAseq” show sequence coverage in our RNA-seq experiments. Each ChIP-seq and RNA-seq coverage track is scaled individually, according to the maximum coverage in that dataset within the viewing window; scales for each track can be seen on the sidebar. The three tracks with labels ending “_RNAseq_splice_junctions” show the number of spliced reads supporting each predicted splice junction, with junctions supported by only 1–10 reads in gray, junctions supported by 11–99 reads in orange, and those supported by ≥100 reads in red.
(PDF)

Figure S10 UCSC browser screenshot showing details of THE1C-lncRNA genomic region, including RNA-seq data. We use the UCSC Genome Browser [88] to display locations of repetitive elements and genes, and use custom tracks to show various additional features and as well as data generated in our lab. We created the blue and red tracks labeled “LTRs_showing_type” and “LTR_internal_regions_showing_type” by filtering UCSC's RepeatMasker track so that only LTR-type repeats are shown (blue, only the long terminal repeats; red, only the internal regions), along with a label for each repeat that shows the repeat family and subtype. The pink “DUX4_ChIPseq” track shows fragment coverage in our ChIP-seq experiment, and the “DUX4_ChIPseq_peaks” track shows the 63,795 peaks called from that coverage data. DUX4 motifs show any 17-mer sequence matching the DUX4 PWM with score of ≥9.75 (see Methods), labeled by score. We also include a track showing locations of the primer sequences given in Table S6. The six black tracks with labels ending “_RNAseq” show sequence coverage in our RNA-seq experiments. Each ChIP-seq and RNA-seq coverage track is scaled individually, according to the maximum coverage in that dataset within the viewing window; scales for each track can be seen on the sidebar. The three tracks with labels ending “_RNAseq_splice_junctions” show the number of spliced reads supporting each predicted splice junction, with junctions supported by only 1–10 reads in gray, junctions supported by 11–99 reads in orange, and those supported by ≥100 reads in red.
(PDF)

Figure S11 UCSC browser screenshot showing details of DDX10-antisense genomic region, including RNA-seq data. We use the UCSC Genome Browser [88] to display locations of repetitive elements and genes, and use custom tracks to show various additional features and as well as data generated in our lab. We created the blue and red tracks labeled “LTRs_showing_type” and “LTR_internal_regions_showing_type” by filtering UCSC's RepeatMasker track so that only LTR-type repeats are shown (blue, only the long terminal repeats; red, only the internal regions), along with a label for each repeat that shows the repeat family and subtype. The pink “DUX4_ChIPseq” track shows fragment coverage in our ChIP-seq experiment, and the “DUX4_ChIPseq_peaks” track shows the 63,795 peaks called from that coverage data. DUX4 motifs show any 17-mer sequence matching the DUX4 PWM with score of ≥9.75 (see Methods), labeled by score. We also include a track showing locations of the primer sequences given in Table S6. The six black tracks with labels ending “_RNAseq” show sequence coverage in our RNA-seq experiments. Each ChIP-seq and RNA-seq coverage track is scaled individually, according to the maximum coverage in that dataset within the viewing window; scales for each track can be seen on the sidebar. The three tracks with labels ending “_RNAseq_splice_junctions” show the number of spliced reads supporting each predicted splice junction, with junctions supported by only 1–10 reads in gray, junctions supported by 11–99 reads in orange, and those supported by ≥100 reads in red.
(PDF)

Figure S12 Multiple variants of the HSATII repeat are expressed. We aligned HSATII RNA-seq reads to the HSATII consensus sequence (see Methods), and used the IGV browser [87] to display the resulting alignment. In the narrow bottom panel of the display, each nucleotide of the consensus sequence is represented by a colored tick mark (green = A; blue = C; brown = G; red = T). The next two panels from the bottom represent two alignments, each showing RNA-seq data from a different *DUX4*-transduced myoblast cell line (upper alignment, MB135 cells, 1,182,329 aligned reads; lower alignment, 54-1 cells, 288,741 aligned reads). In the alignments, each sequence read is shown as a very thin gray line, stacked densely on top of one another. In many regions of the alignment where coverage is very deep, IGV displays only a subset of reads for enhanced visibility. IGV shows positions in each sequence read that do not match the consensus sequence as small colored tick marks, color coded (green = A; blue = C; brown = G; red = T; black = deletion; purple = insertion). It is clear from the number and diversity of non-reference bases among the aligned reads that multiple HSATII variants are transcribed.
(PDF)

Table S1 DUX4-binding site locations in the hg19 version of the human genome assembly. The “repetitive element” column shows whether the peak's best DUX4 motif is in unique sequence, or if not what kind of repetitive element it is in. The last two columns show results from differential expression analysis of the peak plus 1 kb of flanking sequence on each side, comparing expression levels in myoblasts transduced with lentivirus carrying *DUX4* to control myoblasts.
(XLSX)

Table S2 Enrichment estimates for each repeat type among ChIP-seq peaks, ChIP-seq reads and RNA-seq reads. Repeat types are shown sorted by peak-based enrichment estimate. See legend to Table 1. Tables S2 and S3 additionally include peak enrichment estimates calculated by comparing observed proportions of peaks overlapping each repeat type to the repeat content of randomly sampled AT-content-matched 17-mers (see Methods).
(XLSX)

Table S3 Enrichment estimates for each repeat family among ChIP-seq peaks, ChIP-seq reads and RNA-seq reads. Repeat families are shown sorted by peak-based enrichment estimate.
(XLSX)

Table S4 DUX4-bound regions used as previously undescribed alternative promoters for annotated genes (see Methods). We note that *HEY1* does not appear in this list for technical reasons, because several intervening exons are spliced between the DUX4-bound repeat and the annotated gene, so that no 100 bp sequencing read directly joins the repeat and *HEY1*.
(XLSX)

Table S5 DUX4-bound regions used as previously undescribed alternative promoters for annotated lncRNAs (see Methods).
(XLSX)

Table S6 Primers used for RT-PCR and 5′-RACE experiments.
(XLSX)
