# Supplementary figures and images for: DUX4 Binding to Retroelements Creates Promoters That Are Active in FSHD Muscle and Testis
Source: PLoS Genet. 2013 Nov 21;9(11):e1003947. doi: 10.1371/journal.pgen.1003947 (PMC3836709; doi:10.1371/journal.pgen.1003947)

Supplementary Figure 1

**A**

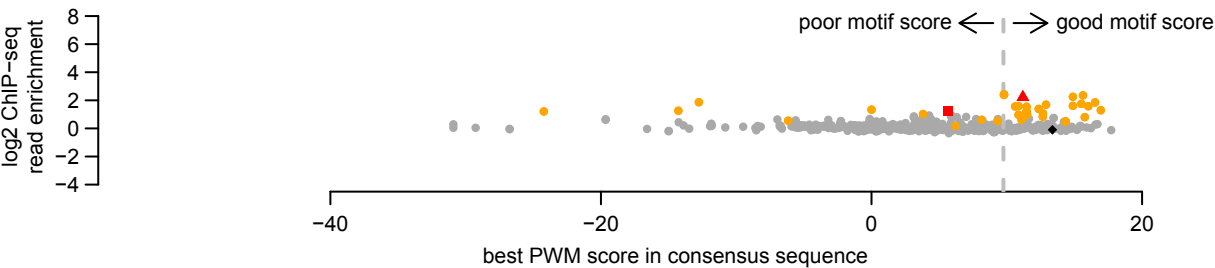

**B**

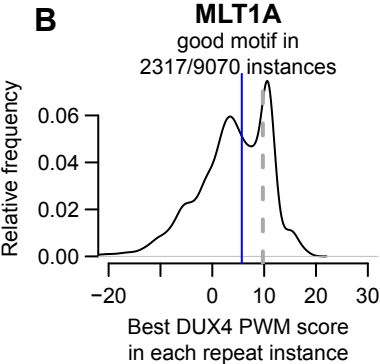

**C**

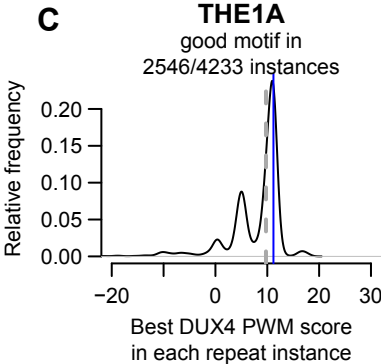

**D**

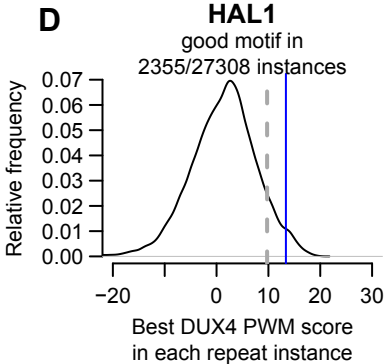

Supplement: Figure S1 — Consensus sequences for most bound repeat types contain a DUX4 binding motif. We scanned repeat consensus sequences with a PWM representing DUX4's binding preferences. In all panels, the dashed gray vertical line represents an ad hoc PWM score threshold of 9.75: most ChIP-seq peaks exceed this threshold. (A) For each repeat type, we plot read-based ChIP-seq enrichment estimate (y-axis) against best PWM score in the consensus (x-axis). We show only repeats with ≥1000 mapped reads, because enrichment estimates derived from fewer reads are less biologically significant and more error-prone. Orange and red datapoints show repeats enriched (≥2-fold) among DUX4 binding sites by either the peak-based (≥100 peaks) or the read-based method (≥1000 reads); most contain a good DUX4 motif in their consensus sequence. To explore exceptions, we investigated three repeat types: MLT1A (red square, and panel B); THE1A (red triangle, and panel C);and HAL1 (black diamond, and panel D). (B, C, D) Distribution of best PWM scores for every genomic instance of each repeat type, demonstrating that consensus motif scores may not represent genomic instances well. Blue vertical lines show best consensus motif scores. Text below each title shows how many genomic instances of the repeat contain ≥1 motif scoring ≥9.75. (B) MLT1A repeats are enriched among DUX4 binding sites. Although their consensus has no good motif, a reasonable proportion of genomic repeat instances do, explaining DUX4 binding. (C) Some repeats, like THE1As, have a good motif in their consensus and are enriched for DUX4 binding; many genomic instances contain a motif identical to that of the consensus sequence. (D) Other repeats, like HAL1s, have a good motif in their consensus but only in a few genomic instances, explaining the lack of DUX4 binding. Such discrepancies between consensus PWM scores and repeat instances can occur because repeat instances acquire post-insertion mutations, and/or because of inaccuracy in the conse [file pgen.1003947.s002.pdf]

**Supplementary Figure 2**

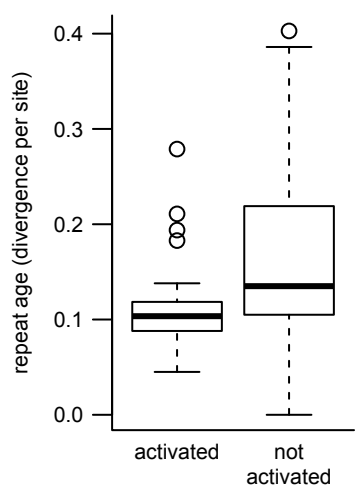

Supplement: Figure S2 — Activated internal repeats are younger than those that do not show activation. Repeat age is estimated using divergence per site from consensus sequence as a proxy (as reported by RepeatMasker). We show divergence for a filtered dataset of the internal regions of LTR-type elements that are close to DUX4-bound LTRs, showing those that are transcriptionally activated to a statistically significant level (“activated”) separately from the remaining elements (“not activated”). The activated elements show lower divergence from consensus sequences (i.e. are younger) than the remaining elements. (PDF) [file pgen.1003947.s003.pdf]

Supplementary Figure 3

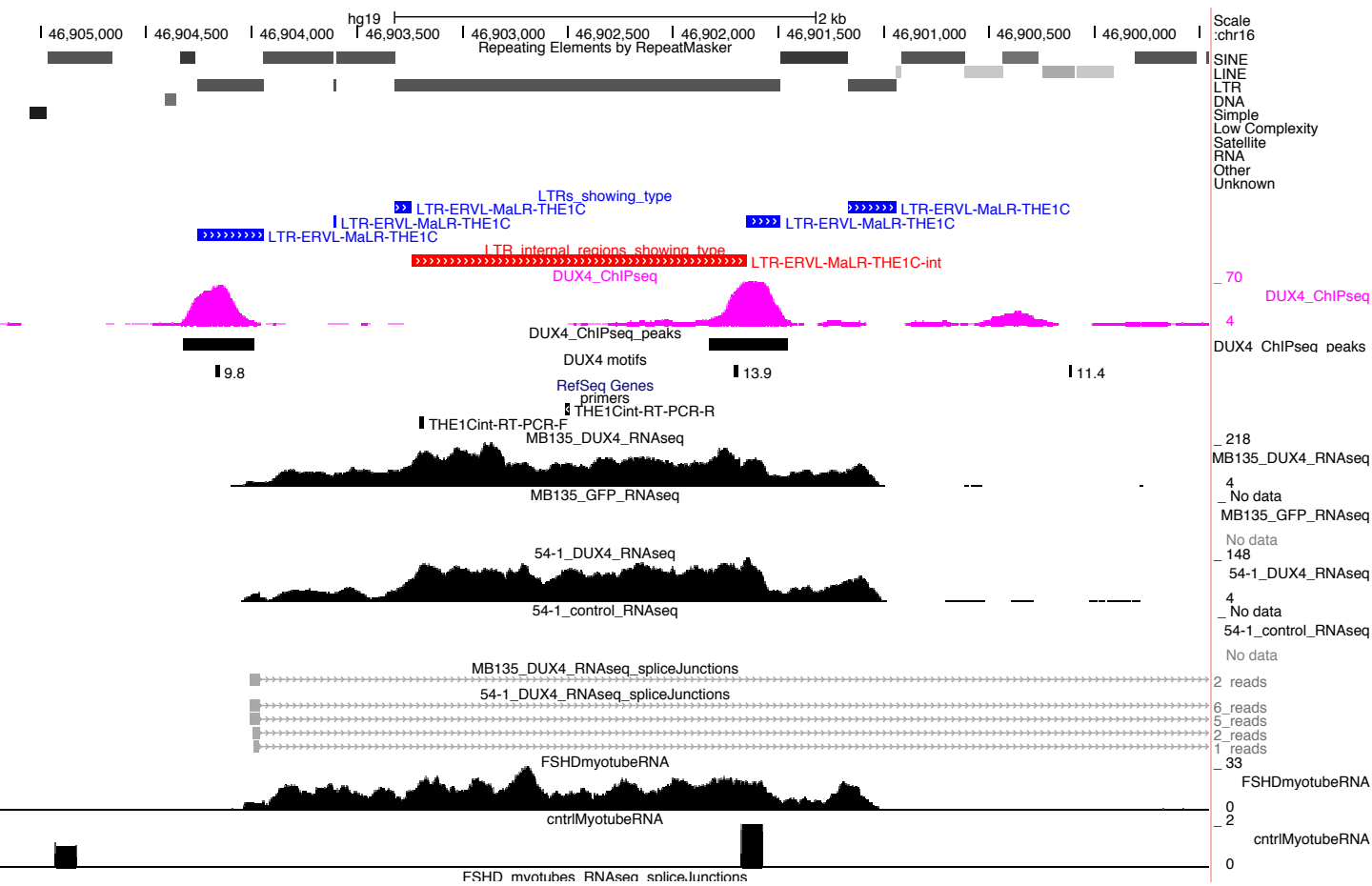

Supplement: Figure S3 — UCSC browser screenshot showing details of THE1C genomic region, including RNA-seq data. We use the UCSC Genome Browser [88] to display locations of repetitive elements and genes, and use custom tracks to show various additional features and as well as data generated in our lab. We created the blue and red tracks labeled “LTRs_showing_type” and “LTR_internal_regions_showing_type” by filtering UCSC's RepeatMasker track so that only LTR-type repeats are shown (blue, only the long terminal repeats; red, only the internal regions), along with a label for each repeat that shows the repeat family and subtype. The pink “DUX4_ChIPseq” track shows fragment coverage in our ChIP-seq experiment, and the “DUX4_ChIPseq_peaks” track shows the 63,795 peaks called from that coverage data. DUX4 motifs show any 17-mer sequence matching the DUX4 PWM with score of ≥9.75 (see Methods), labeled by score. We also include a track showing locations of the primer sequences given in Table S6. The six black tracks with labels ending “_RNAseq” show sequence coverage in our RNA-seq experiments. Each ChIP-seq and RNA-seq coverage track is scaled individually, according to the maximum coverage in that dataset within the viewing window; scales for each track can be seen on the sidebar (e.g. coverage shown for the DUX4_ChIPseq track ranges from 4–70, but ranges from 4–218 for the MB135_DUX4_RNAseq track). The three tracks with labels ending “_RNAseq_splice_junctions” show the number of spliced reads supporting each predicted splice junction, with junctions supported by only 1–10 reads in gray, junctions supported by 11–99 reads in orange, and those supported by ≥100 reads in red. (PDF) [file pgen.1003947.s004.pdf]

Supplementary Figure 4

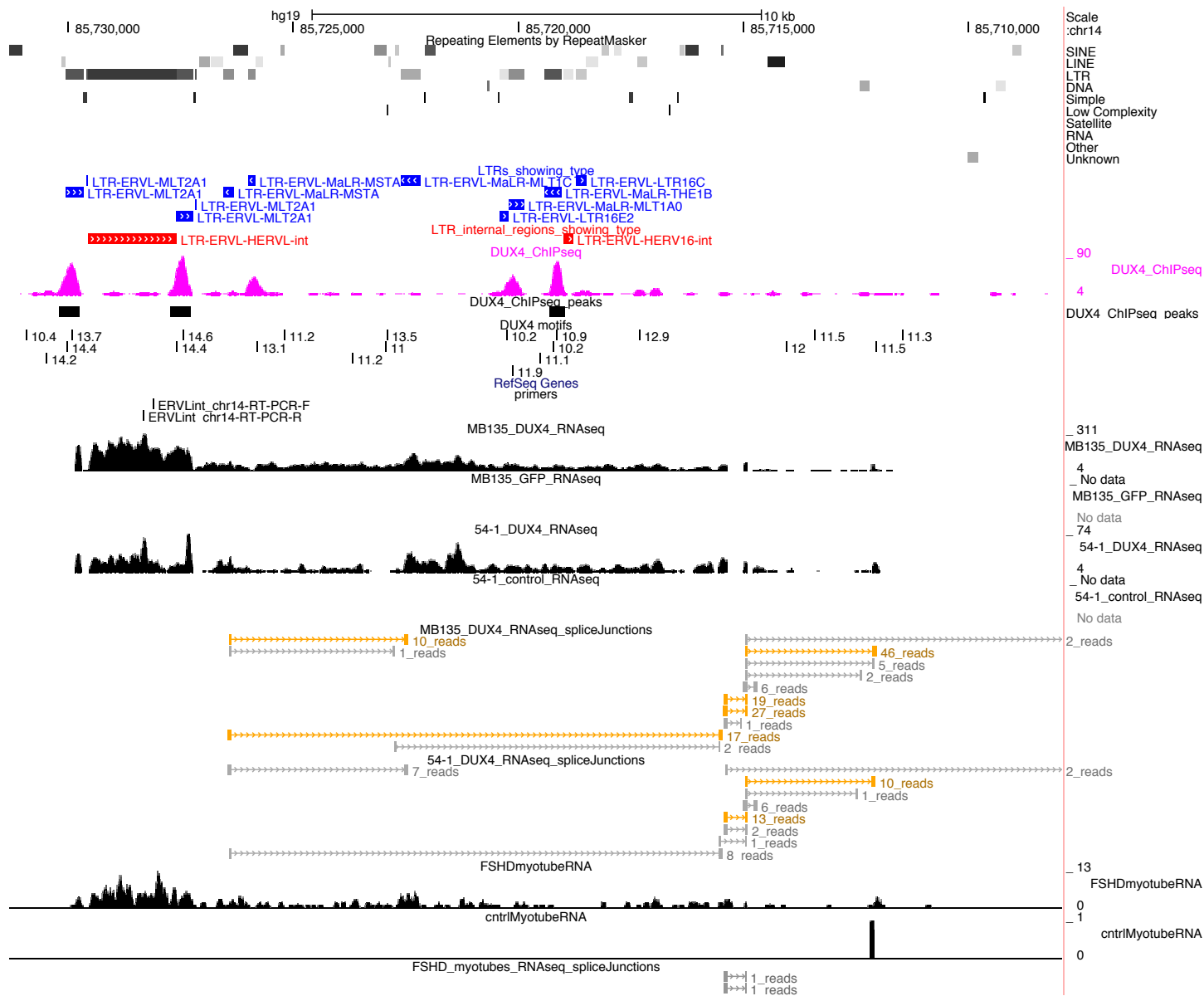

Supplement: Figure S4 — UCSC browser screenshot showing details of ERVL genomic region on chromosome 14, including RNA-seq data. We use the UCSC Genome Browser [88] to display locations of repetitive elements and genes, and use custom tracks to show various additional features and as well as data generated in our lab. We created the blue and red tracks labeled “LTRs_showing_type” and “LTR_internal_regions_showing_type” by filtering UCSC's RepeatMasker track so that only LTR-type repeats are shown (blue, only the long terminal repeats; red, only the internal regions), along with a label for each repeat that shows the repeat family and subtype. The pink “DUX4_ChIPseq” track shows fragment coverage in our ChIP-seq experiment, and the “DUX4_ChIPseq_peaks” track shows the 63,795 peaks called from that coverage data. DUX4 motifs show any 17-mer sequence matching the DUX4 PWM with score of ≥9.75 (see Methods), labeled by score. We also include a track showing locations of the primer sequences given in Table S6. The six black tracks with labels ending “_RNAseq” show sequence coverage in our RNA-seq experiments. Each ChIP-seq and RNA-seq coverage track is scaled individually, according to the maximum coverage in that dataset within the viewing window; scales for each track can be seen on the sidebar. The three tracks with labels ending “_RNAseq_splice_junctions” show the number of spliced reads supporting each predicted splice junction, with junctions supported by only 1–10 reads in gray, junctions supported by 11–99 reads in orange, and those supported by ≥100 reads in red. (PDF) [file pgen.1003947.s005.pdf]

Supplementary Figure 5

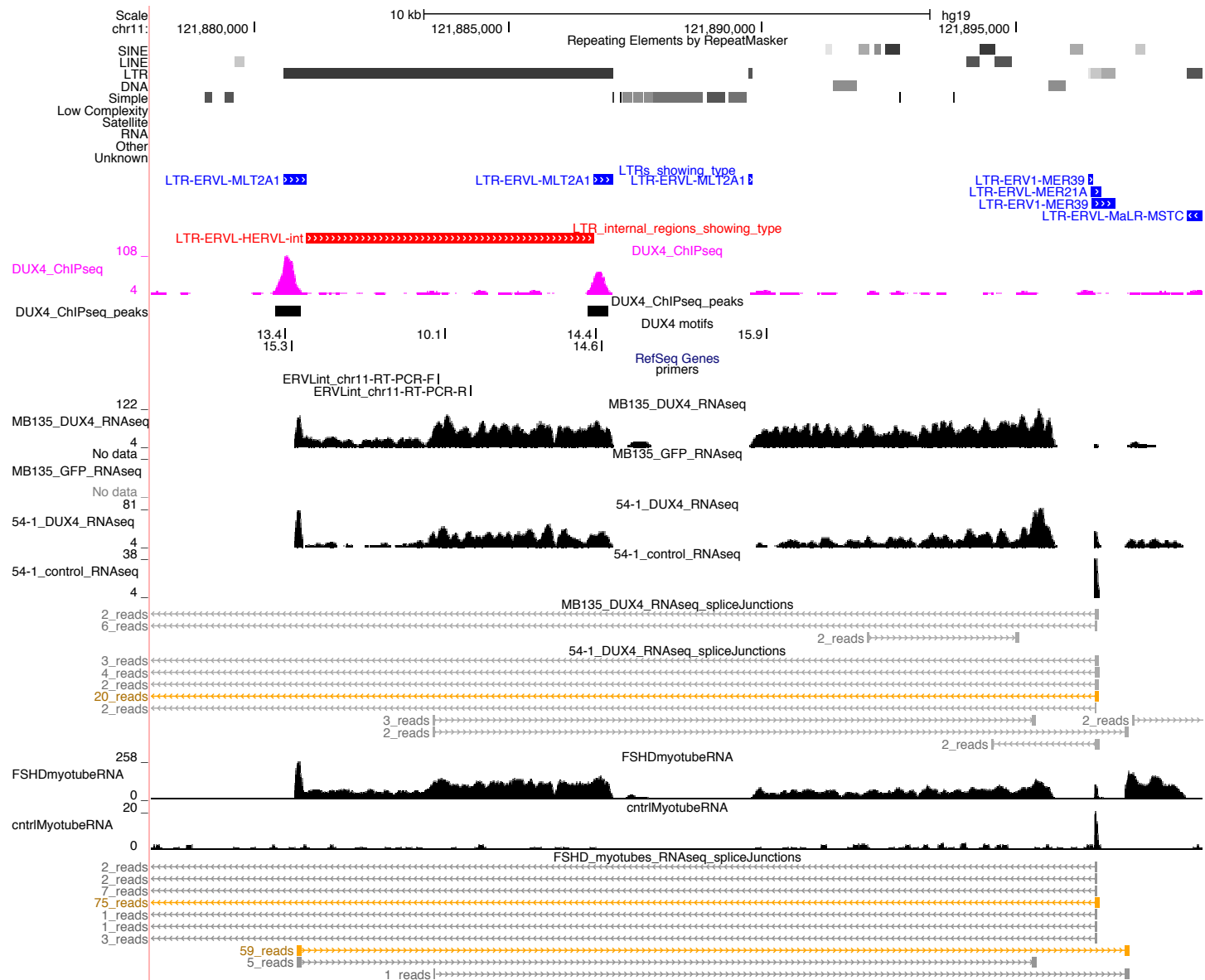

Supplement: Figure S5 — UCSC browser screenshot showing details of ERVL genomic region on chromosome 11, including RNA-seq data. We use the UCSC Genome Browser [88] to display locations of repetitive elements and genes, and use custom tracks to show various additional features and as well as data generated in our lab. We created the blue and red tracks labeled “LTRs_showing_type” and “LTR_internal_regions_showing_type” by filtering UCSC's RepeatMasker track so that only LTR-type repeats are shown (blue, only the long terminal repeats; red, only the internal regions), along with a label for each repeat that shows the repeat family and subtype. The pink “DUX4_ChIPseq” track shows fragment coverage in our ChIP-seq experiment, and the “DUX4_ChIPseq_peaks” track shows the 63,795 peaks called from that coverage data. DUX4 motifs show any 17-mer sequence matching the DUX4 PWM with score of ≥9.75 (see Methods), labeled by score. We also include a track showing locations of the primer sequences given in Table S6. The six black tracks with labels ending “_RNAseq” show sequence coverage in our RNA-seq experiments. Each ChIP-seq and RNA-seq coverage track is scaled individually, according to the maximum coverage in that dataset within the viewing window; scales for each track can be seen on the sidebar. The three tracks with labels ending “_RNAseq_splice_junctions” show the number of spliced reads supporting each predicted splice junction, with junctions supported by only 1–10 reads in gray, junctions supported by 11–99 reads in orange, and those supported by ≥100 reads in red. (PDF) [file pgen.1003947.s006.pdf]

Supplementary Figure 6

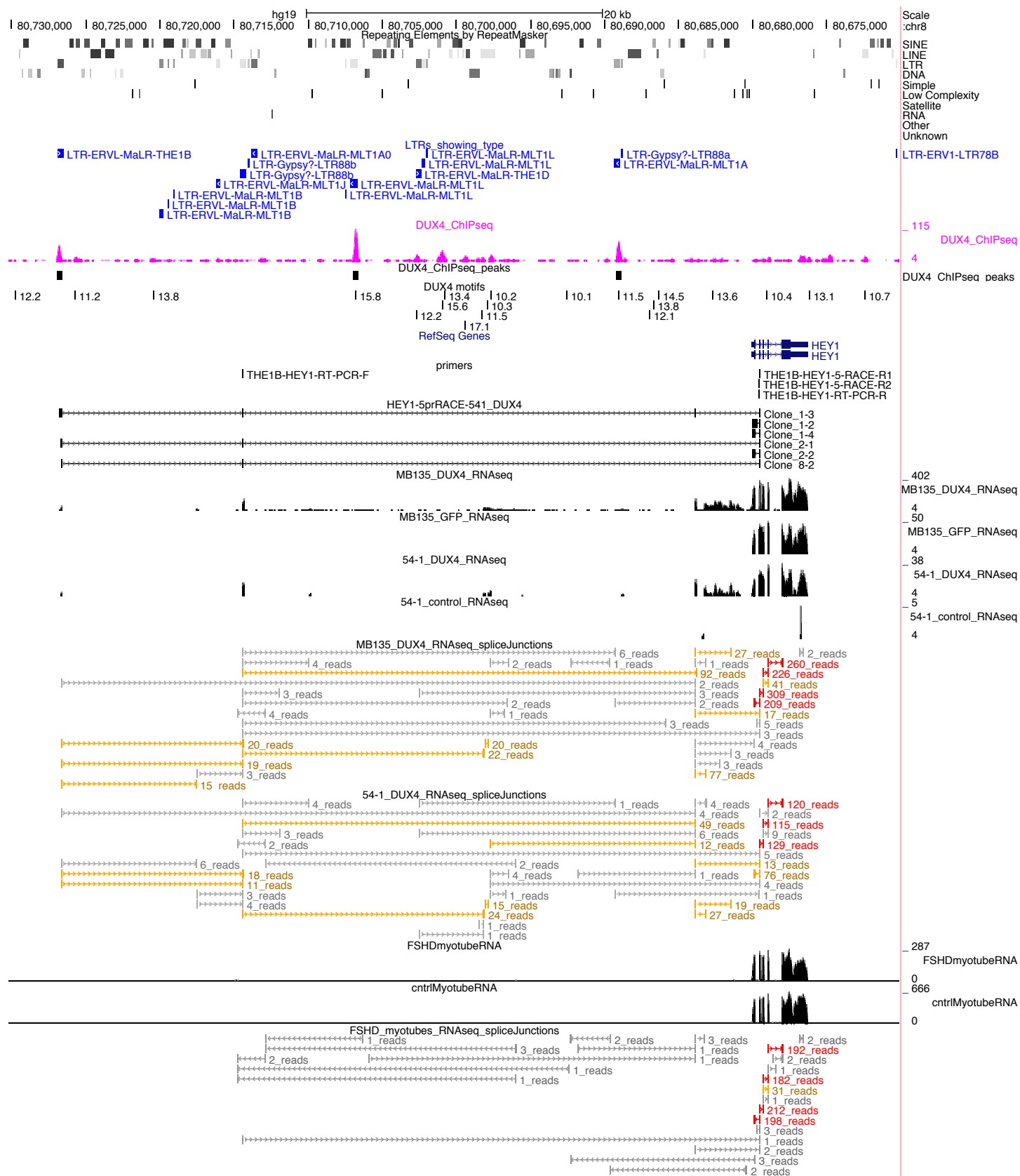

Supplement: Figure S6 — UCSC browser screenshot showing details of HEY1 genomic region, including RNA-seq data. We use the UCSC Genome Browser [88] to display locations of repetitive elements and genes, and use custom tracks to show various additional features and as well as data generated in our lab. We created the blue and red tracks labeled “LTRs_showing_type” and “LTR_internal_regions_showing_type” by filtering UCSC's RepeatMasker track so that only LTR-type repeats are shown (blue, only the long terminal repeats; red, only the internal regions), along with a label for each repeat that shows the repeat family and subtype. The pink “DUX4_ChIPseq” track shows fragment coverage in our ChIP-seq experiment, and the “DUX4_ChIPseq_peaks” track shows the 63,795 peaks called from that coverage data. DUX4 motifs show any 17-mer sequence matching the DUX4 PWM with score of ≥9.75 (see Methods), labeled by score. We also include a track showing locations of the primer sequences given in Table S6, and a track that shows sequences of 5′ RACE products. The six black tracks with labels ending “_RNAseq” show sequence coverage in our RNA-seq experiments. Each ChIP-seq and RNA-seq coverage track is scaled individually, according to the maximum coverage in that dataset within the viewing window; scales for each track can be seen on the sidebar. The three tracks with labels ending “_RNAseq_splice_junctions” show the number of spliced reads supporting each predicted splice junction, with junctions supported by only 1–10 reads in gray, junctions supported by 11–99 reads in orange, and those supported by ≥100 reads in red. (PDF) [file pgen.1003947.s007.pdf]

Supplementary Figure 7

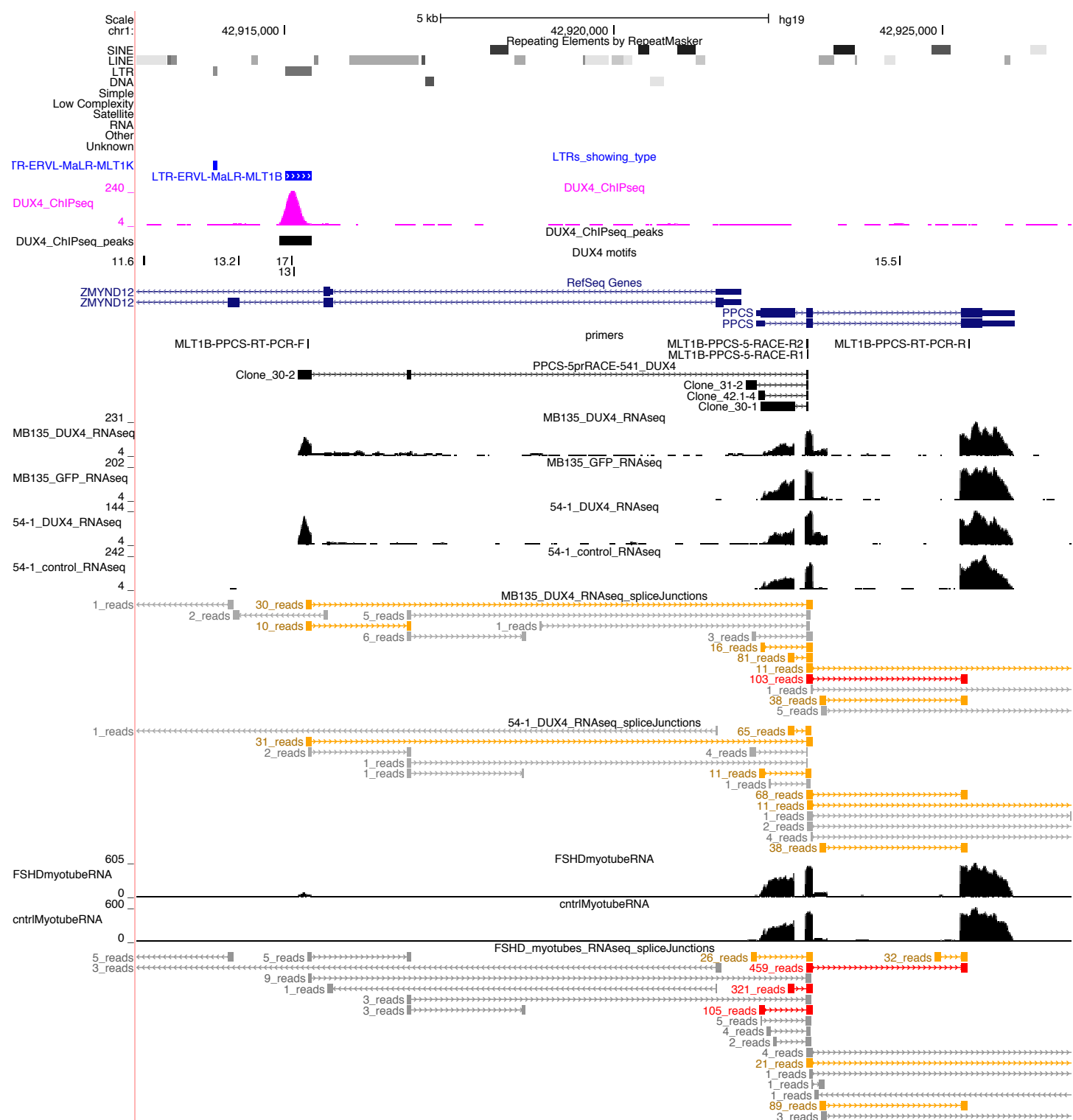

Supplement: Figure S7 — UCSC browser screenshot showing details of PPCS genomic region, including RNA-seq data. We use the UCSC Genome Browser [88] to display locations of repetitive elements and genes, and use custom tracks to show various additional features and as well as data generated in our lab. We created the blue and red tracks labeled “LTRs_showing_type” and “LTR_internal_regions_showing_type” by filtering UCSC's RepeatMasker track so that only LTR-type repeats are shown (blue, only the long terminal repeats; red, only the internal regions), along with a label for each repeat that shows the repeat family and subtype. The pink “DUX4_ChIPseq” track shows fragment coverage in our ChIP-seq experiment, and the “DUX4_ChIPseq_peaks” track shows the 63,795 peaks called from that coverage data. DUX4 motifs show any 17-mer sequence matching the DUX4 PWM with score of ≥9.75 (see Methods), labeled by score. We also include a track showing locations of the primer sequences given in Table S6, and a track that shows sequences of 5′ RACE products. The six black tracks with labels ending “_RNAseq” show sequence coverage in our RNA-seq experiments. Each ChIP-seq and RNA-seq coverage track is scaled individually, according to the maximum coverage in that dataset within the viewing window; scales for each track can be seen on the sidebar. The three tracks with labels ending “_RNAseq_splice_junctions” show the number of spliced reads supporting each predicted splice junction, with junctions supported by only 1–10 reads in gray, junctions supported by 11–99 reads in orange, and those supported by ≥100 reads in red. (PDF) [file pgen.1003947.s008.pdf]

Supplementary Figure 8

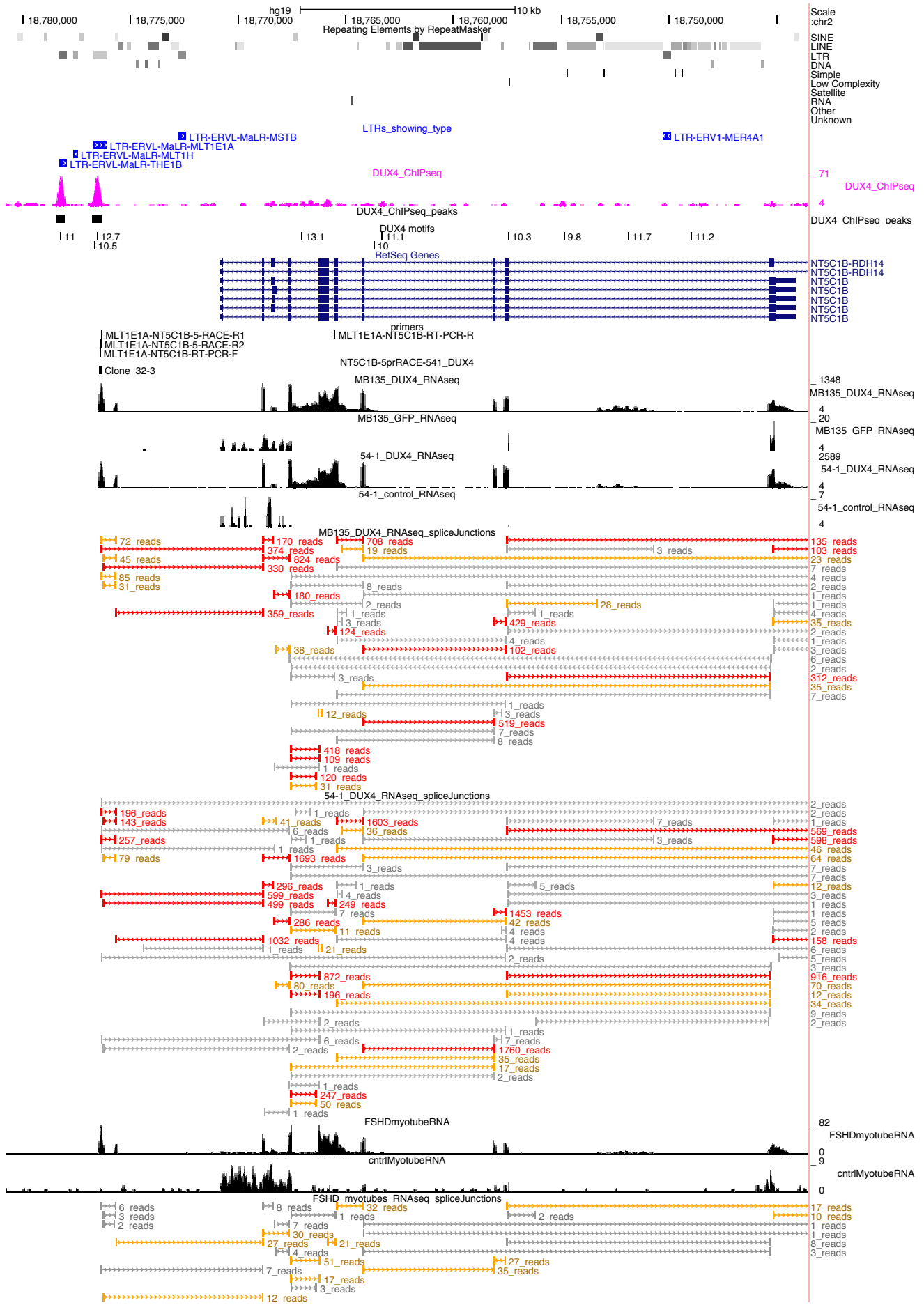

Supplement: Figure S8 — UCSC browser screenshot showing details of NT5C1B genomic region, including RNA-seq data. We use the UCSC Genome Browser [88] to display locations of repetitive elements and genes, and use custom tracks to show various additional features and as well as data generated in our lab. We created the blue and red tracks labeled “LTRs_showing_type” and “LTR_internal_regions_showing_type” by filtering UCSC's RepeatMasker track so that only LTR-type repeats are shown (blue, only the long terminal repeats; red, only the internal regions), along with a label for each repeat that shows the repeat family and subtype. The pink “DUX4_ChIPseq” track shows fragment coverage in our ChIP-seq experiment, and the “DUX4_ChIPseq_peaks” track shows the 63,795 peaks called from that coverage data. DUX4 motifs show any 17-mer sequence matching the DUX4 PWM with score of ≥9.75 (see Methods), labeled by score. We also include a track showing locations of the primer sequences given in Table S6, and a track that shows sequences of 5′ RACE products. The six black tracks with labels ending “_RNAseq” show sequence coverage in our RNA-seq experiments. Each ChIP-seq and RNA-seq coverage track is scaled individually, according to the maximum coverage in that dataset within the viewing window; scales for each track can be seen on the sidebar. The three tracks with labels ending “_RNAseq_splice_junctions” show the number of spliced reads supporting each predicted splice junction, with junctions supported by only 1–10 reads in gray, junctions supported by 11–99 reads in orange, and those supported by ≥100 reads in red. (PDF) [file pgen.1003947.s009.pdf]

Supplementary Figure 9

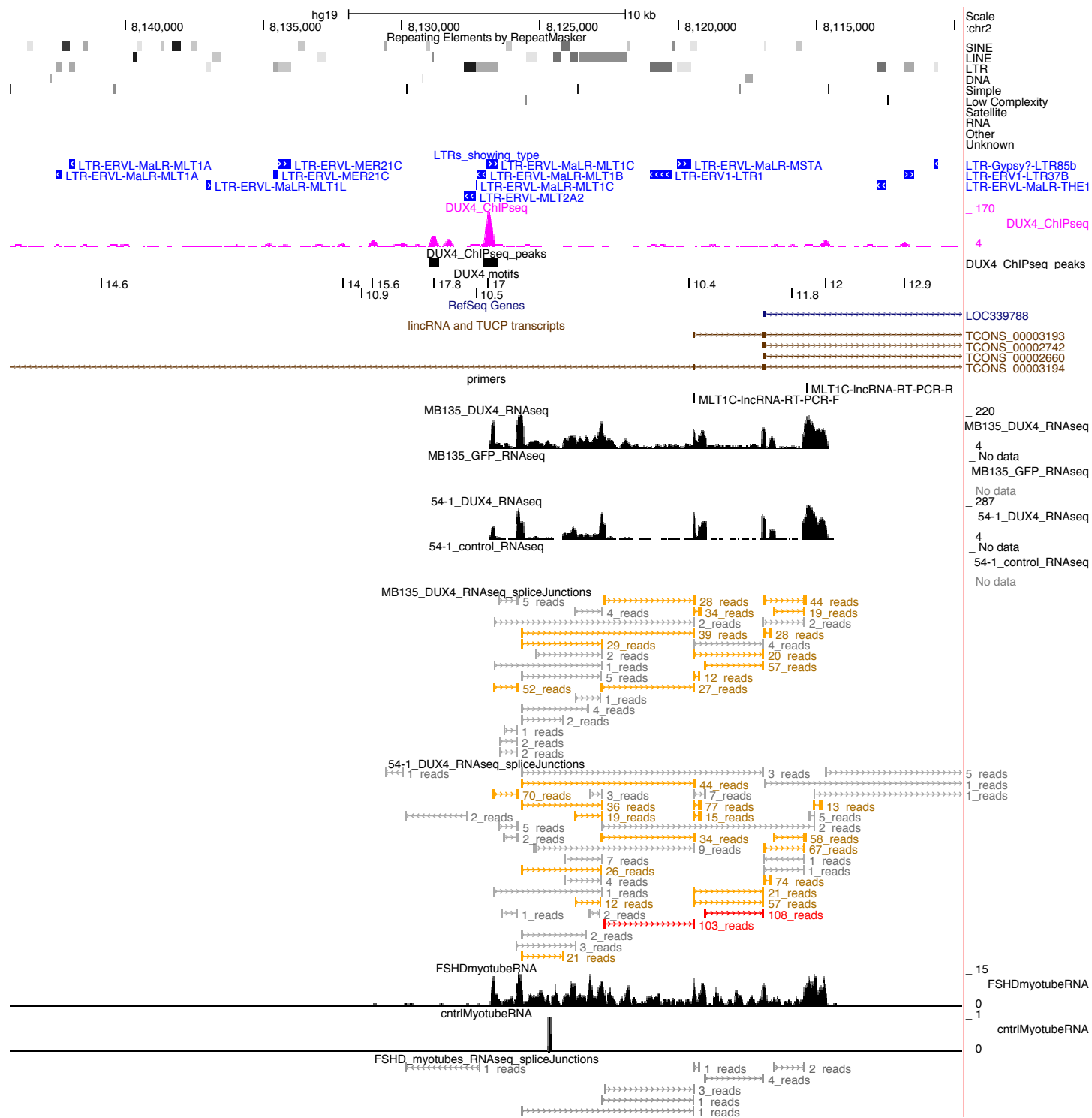

Supplement: Figure S9 — UCSC browser screenshot showing details of MLT1C-lncRNA genomic region, including RNA-seq data. We use the UCSC Genome Browser [88] to display locations of repetitive elements and genes, and use custom tracks to show various additional features and as well as data generated in our lab. We created the blue and red tracks labeled “LTRs_showing_type” and “LTR_internal_regions_showing_type” by filtering UCSC's RepeatMasker track so that only LTR-type repeats are shown (blue, only the long terminal repeats; red, only the internal regions), along with a label for each repeat that shows the repeat family and subtype. The pink “DUX4_ChIPseq” track shows fragment coverage in our ChIP-seq experiment, and the “DUX4_ChIPseq_peaks” track shows the 63,795 peaks called from that coverage data. DUX4 motifs show any 17-mer sequence matching the DUX4 PWM with score of ≥9.75 (see Methods), labeled by score. We also include a track showing locations of the primer sequences given in Table S6. The six black tracks with labels ending “_RNAseq” show sequence coverage in our RNA-seq experiments. Each ChIP-seq and RNA-seq coverage track is scaled individually, according to the maximum coverage in that dataset within the viewing window; scales for each track can be seen on the sidebar. The three tracks with labels ending “_RNAseq_splice_junctions” show the number of spliced reads supporting each predicted splice junction, with junctions supported by only 1–10 reads in gray, junctions supported by 11–99 reads in orange, and those supported by ≥100 reads in red. (PDF) [file pgen.1003947.s010.pdf]

Supplementary Figure 10

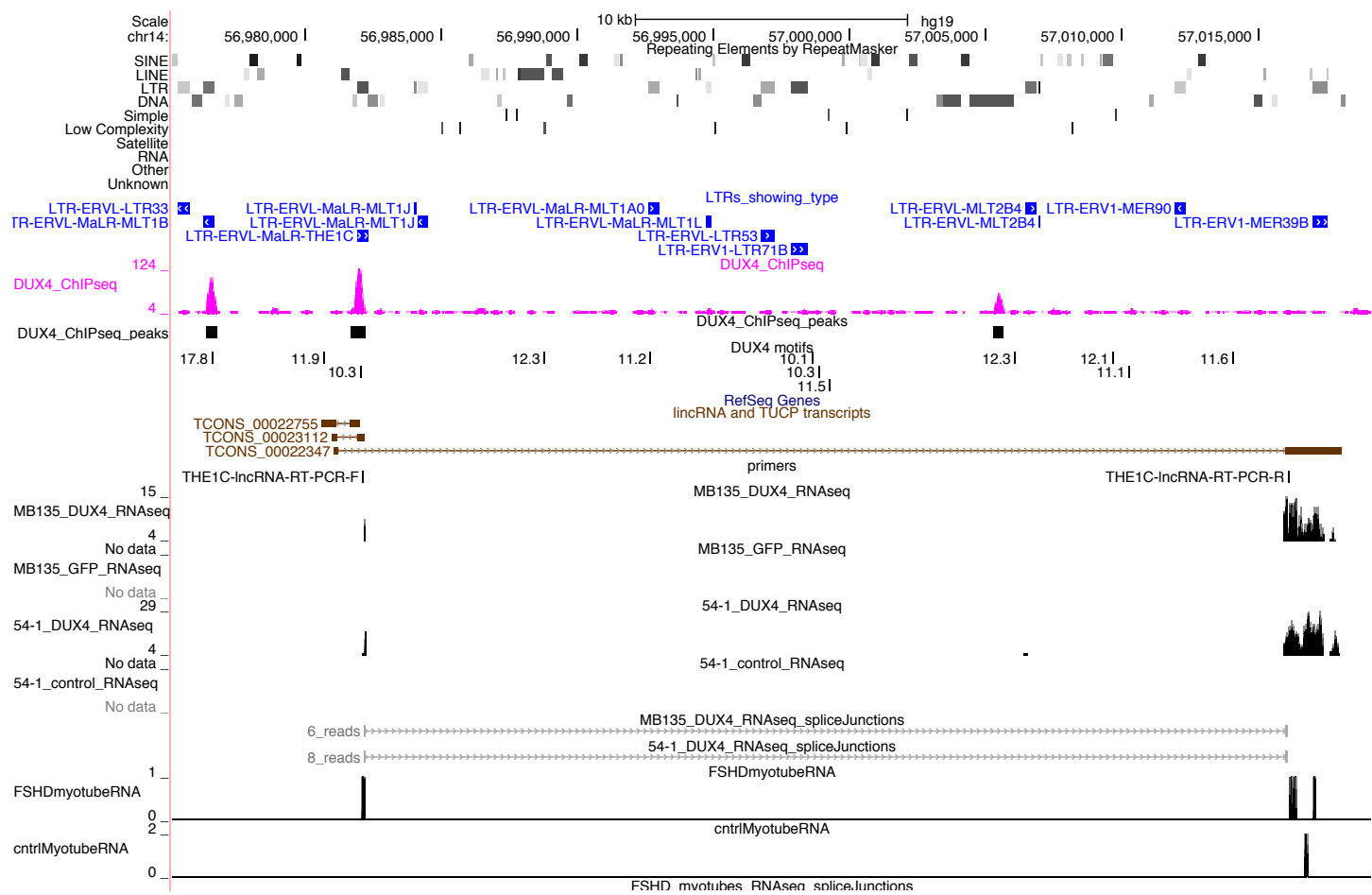

Supplement: Figure S10 — UCSC browser screenshot showing details of THE1C-lncRNA genomic region, including RNA-seq data. We use the UCSC Genome Browser [88] to display locations of repetitive elements and genes, and use custom tracks to show various additional features and as well as data generated in our lab. We created the blue and red tracks labeled “LTRs_showing_type” and “LTR_internal_regions_showing_type” by filtering UCSC's RepeatMasker track so that only LTR-type repeats are shown (blue, only the long terminal repeats; red, only the internal regions), along with a label for each repeat that shows the repeat family and subtype. The pink “DUX4_ChIPseq” track shows fragment coverage in our ChIP-seq experiment, and the “DUX4_ChIPseq_peaks” track shows the 63,795 peaks called from that coverage data. DUX4 motifs show any 17-mer sequence matching the DUX4 PWM with score of ≥9.75 (see Methods), labeled by score. We also include a track showing locations of the primer sequences given in Table S6. The six black tracks with labels ending “_RNAseq” show sequence coverage in our RNA-seq experiments. Each ChIP-seq and RNA-seq coverage track is scaled individually, according to the maximum coverage in that dataset within the viewing window; scales for each track can be seen on the sidebar. The three tracks with labels ending “_RNAseq_splice_junctions” show the number of spliced reads supporting each predicted splice junction, with junctions supported by only 1–10 reads in gray, junctions supported by 11–99 reads in orange, and those supported by ≥100 reads in red. (PDF) [file pgen.1003947.s011.pdf]

Supplementary Figure 11

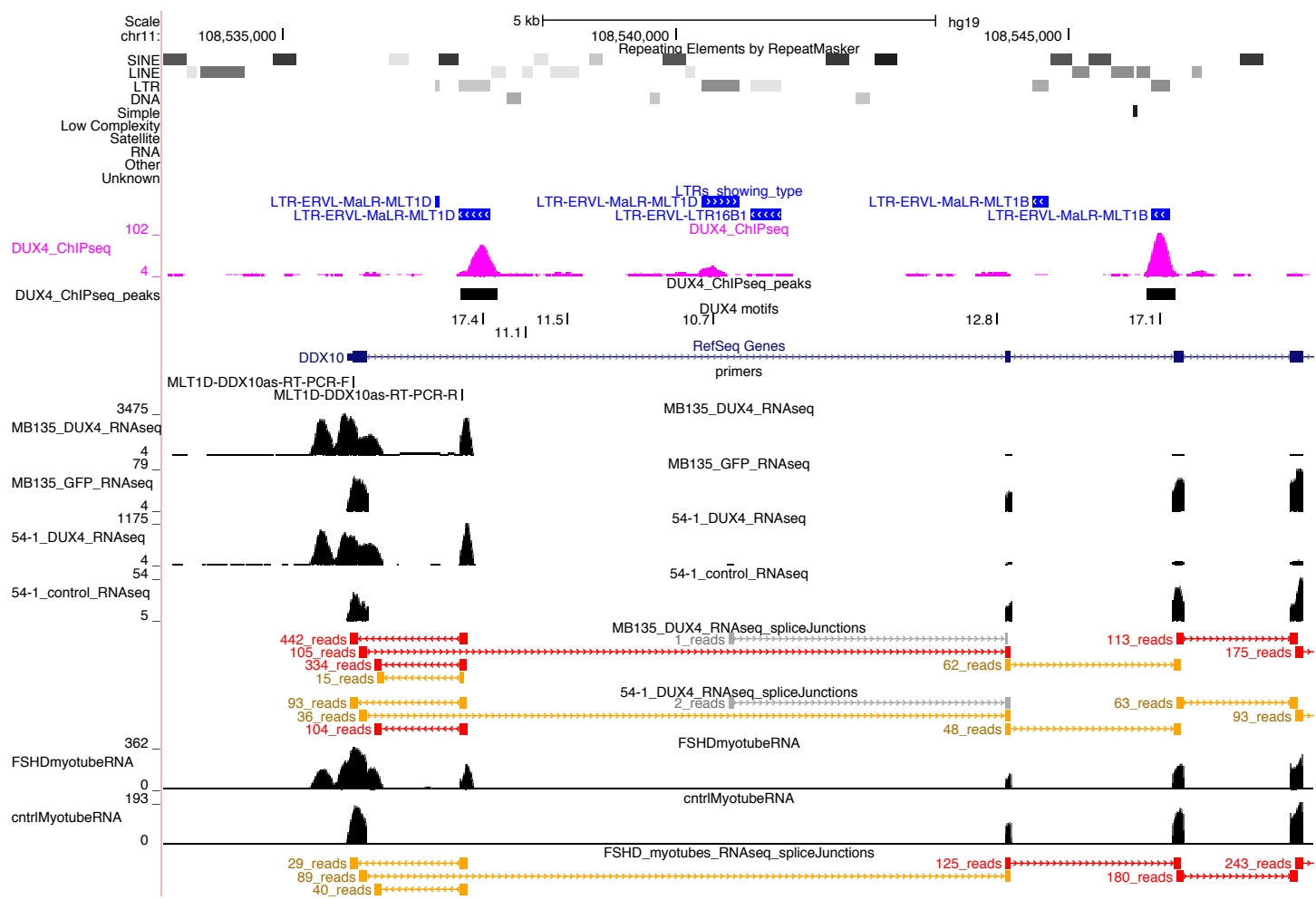

Supplement: Figure S11 — UCSC browser screenshot showing details of DDX10-antisense genomic region, including RNA-seq data. We use the UCSC Genome Browser [88] to display locations of repetitive elements and genes, and use custom tracks to show various additional features and as well as data generated in our lab. We created the blue and red tracks labeled “LTRs_showing_type” and “LTR_internal_regions_showing_type” by filtering UCSC's RepeatMasker track so that only LTR-type repeats are shown (blue, only the long terminal repeats; red, only the internal regions), along with a label for each repeat that shows the repeat family and subtype. The pink “DUX4_ChIPseq” track shows fragment coverage in our ChIP-seq experiment, and the “DUX4_ChIPseq_peaks” track shows the 63,795 peaks called from that coverage data. DUX4 motifs show any 17-mer sequence matching the DUX4 PWM with score of ≥9.75 (see Methods), labeled by score. We also include a track showing locations of the primer sequences given in Table S6. The six black tracks with labels ending “_RNAseq” show sequence coverage in our RNA-seq experiments. Each ChIP-seq and RNA-seq coverage track is scaled individually, according to the maximum coverage in that dataset within the viewing window; scales for each track can be seen on the sidebar. The three tracks with labels ending “_RNAseq_splice_junctions” show the number of spliced reads supporting each predicted splice junction, with junctions supported by only 1–10 reads in gray, junctions supported by 11–99 reads in orange, and those supported by ≥100 reads in red. (PDF) [file pgen.1003947.s012.pdf]

Supplementary Figure 12

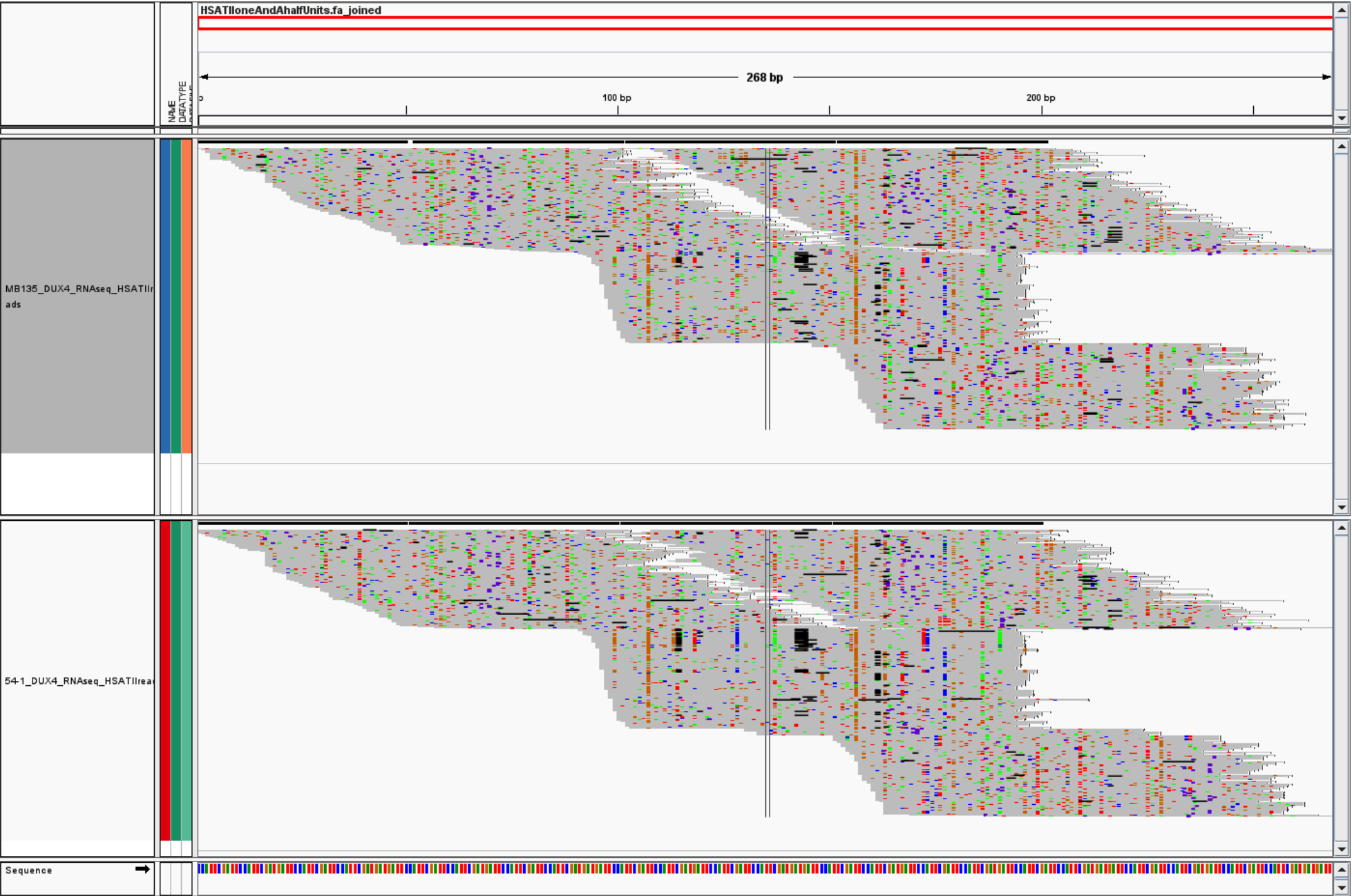

Supplement: Figure S12 — Multiple variants of the HSATII repeat are expressed. We aligned HSATII RNA-seq reads to the HSATII consensus sequence (see Methods), and used the IGV browser [87] to display the resulting alignment. In the narrow bottom panel of the display, each nucleotide of the consensus sequence is represented by a colored tick mark (green = A; blue = C; brown = G; red = T). The next two panels from the bottom represent two alignments, each showing RNA-seq data from a different DUX4-transduced myoblast cell line (upper alignment, MB135 cells, 1,182,329 aligned reads; lower alignment, 54-1 cells, 288,741 aligned reads). In the alignments, each sequence read is shown as a very thin gray line, stacked densely on top of one another. In many regions of the alignment where coverage is very deep, IGV displays only a subset of reads for enhanced visibility. IGV shows positions in each sequence read that do not match the consensus sequence as small colored tick marks, color coded (green = A; blue = C; brown = G; red = T; black = deletion; purple = insertion). It is clear from the number and diversity of non-reference bases among the aligned reads that multiple HSATII variants are transcribed. (PDF) [file pgen.1003947.s013.pdf]
